# Microbial and Chemical Water Quality Assessments Across the Rural and Urban Areas of Nepal: A Scoping Review

**DOI:** 10.3390/ijerph22101526

**Published:** 2025-10-05

**Authors:** Suhana Chattopadhyay, Alex Choiniere, Nedelina Tchangalova, Yunika Acharya, Amy R. Sapkota, Leena Malayil

**Affiliations:** 1 Department of Global, Environmental, and Occupational Health, School of Public Health, University of Maryland, College Park, MD 20742, USA; suhanac@umd.edu (S.C.); achoini1@umd.edu (A.C.); ars@umd.edu (A.R.S.); 2 STEM Library, University of Maryland, College Park, MD 20742, USA; nedelina@umd.edu; 3 Research and Development Division, Dhulikhel Hospital, Dhulikhel 45210, Nepal; yunika@dhulikhelhospital.org

**Keywords:** environmental monitoring, water microbiology, water pollutants, arsenic poisoning, metal poisoning, Nepal

## Abstract

Nepal is currently facing critical water quality challenges due to urbanization, water management and governance issues, as well as natural disasters. This has resulted in the presence of harmful contaminants (e.g., pathogens, nitrates, arsenic) across multiple water sources, subsequently leading to waterborne disease risks (e.g., cholera and typhoid). In response to these environmental and public health concerns, we conducted a scoping review to assess microbial and chemical contaminants in drinking and irrigation water in Nepal, as well as their potential impacts on public health. Following the JBI Manual for Evidence Synthesis and the PRISMA-SCR guidelines, we systematically searched for peer-reviewed literature on Nepal’s water quality in seven databases. Of 3666 unique records screened using predefined inclusion criteria, 140 met our criteria. The studies encompassed a variety of methodological designs, with the majority focusing on water sources in the Bagmati province. Bacteria and arsenic emerged as the most prevalent contaminants. Additionally, diseases such as arsenicosis and typhoid remain widespread and may be linked to contaminated water sources. The review identified key gaps in Nepal’s water quality management, including limited geographic research coverage, inconsistent testing protocols, weak regulatory enforcement, and a lack of integration of water quality with public health planning. Our findings underscore the urgent need for effective surveillance systems and a robust regulatory framework to promptly respond to water contamination events in Nepal.

## 1. Introduction

Clean and safe water is essential for maintaining the overall health of a community and its environment. Faced with climate variability and rapid urbanization, low- and middle-income (LMIC) countries remain vulnerable to water scarcity, including Nepal, where only a small percentage of the household population (19%) has access to safely managed drinking water [1], a number that has not improved much over the years [2]. The variable climate exacerbates these water challenges with unpredictable rainfall patterns and glacial melt, affecting the quantity of water available for drinking and irrigation purposes. For example, the Kathmandu Upatyaka Khanepani Limited (KUKL) reported a daily demand of 485 million liters of water in the Kathmandu Valley [3]. In contrast, the available amount was only 175.02 million liters per day, with an average supply of 129.63 million liters per day due to a 20% loss [3]. 

Coupled with limited water quantity, access to high-quality, safe drinking and irrigation water sources in Nepal varies significantly. According to Nepal’s Department of Water Supply and Sewage, it is estimated that although the majority of Nepal’s population of 31.1 million [4] has access to drinking water, this water is not considered ‘safe,’ and significant differences in water quality exist between urban and rural areas [1]. For example, while 21.9% of the household population in the urban region receives safely managed drinking water, the percentage drops to only 13.3% in rural regions [1]. 

In addition to having limited access to safe drinking and irrigation waters, Nepal faces challenges in water quality that differ between urban and rural areas. For example, those living in densely populated areas like the Kathmandu Valley are exposed to greater anthropogenic water contamination, in contrast to those living in rural areas [5]. The rapid growth of the population in urban regions has led to an increase in solid waste production. Limited infrastructure to treat sewage, excessive use of agrochemicals and pesticides in agriculture, and unprotected springs, wells, and surface waters are the major contributors to water pollution in Nepal [6,7]. An estimated 75 tons of waste is dumped into rivers from Kathmandu’s highly populated capital city, leading to a highly contaminated drinking water supply [8,9]. Meanwhile, rural communities, especially in the Terai region, rely on tube wells for drinking water but face chemical contamination from arsenic and nitrates [10]. Other chemical contaminants, like pesticides, frequently contaminate drinking water supplies via agricultural runoff [6]. Besides chemical and heavy metal contamination, Nepal also suffers from widespread microbial contamination in drinking and irrigation water sources [11]. Waterborne diseases such as cholera and typhoid are common, especially in rural areas, with an estimated 817 children dying from diarrhea annually in Nepal [12]. Additionally, these contaminated water sources are frequently used for food crop irrigation, and when irrigated crops are ingested, these exposures could potentially lead to food-borne infections/outbreaks [11]. 

To address these issues, Nepal has set ambitious national targets for water and sanitation by 2030 [13], including 99% coverage for basic water supply and 95% for improved sanitation, aligning with its constitutional recognition of clean water as a fundamental right [14]. To support these goals, the government has developed a comprehensive legislative framework, including National Drinking Water Quality Standards [15], multiple acts regulating water supply and sanitation (The Nepal Water Supply Corporation Act 1989, Local Self Governance Act 1999, and Water Supply and Sanitation Act 2022), and policies promoting environmental sustainability concerning irrigation and wastewater management. These legal instruments emphasize safe drinking water, pollution prevention, and integrated water resource management [16].

Similarly, the United Nations’ Sustainable Development Goal (SDG) 6 aims to achieve “universal access to safe and affordable drinking water for all by 2030” [17]. Since the adoption of the SDG aims in 2016, Nepal has made significant strides in the Water, Sanitation, and Hygiene (WASH) sector, achieving 95.5% sanitation coverage and 88.6% water supply coverage [18]. However, significant knowledge gaps in water quality data and the identification of contamination sources continue to impede further advancement. Identifying these sources is crucial for targeted mitigation efforts, while comprehensive monitoring networks are essential for detecting contamination hotspots. In addition, studies on the health impacts of consuming or using contaminated water are vital for informing the public health care system, designing effective interventions, and raising public awareness. 

Despite the increasing water contamination and lack of access to clean and safe drinking and irrigation water, to our knowledge, there are no systematic or scoping reviews to date that summarize the extent of water contamination, characterize the microbial and chemical contaminants studied in various localities, and evaluate their impacts on community health. Therefore, we conducted a scoping review of peer-reviewed studies on drinking and irrigation waters in Nepal to address these gaps. This methodology allowed us to comprehensively summarize the microbial and chemical contaminants prevalent in the region and assess their potential impact on public health outcomes.

Specifically, our scoping review aimed to identify studies assessing the quality of drinking and irrigation water sources that might potentially impact human health in Nepal. The specific objectives of our study were to: (1) identify and characterize the microbiological quality of drinking and irrigation water in Nepal; (2) understand and evaluate the chemical quality of drinking and irrigation water; and (3) identify the key health outcomes from exposure to microbial and chemical contaminants present in drinking and irrigation water.

## 2. Methods

### 2.1. Protocol and Registration

Following the JBI Manual for Evidence Synthesis [19] and the Preferred Reporting Items for Systematic Reviews and Meta-Analysis for Scoping Reviews (PRISMA-ScP) Guidelines [20], we developed a protocol in advance, defining the objectives, inclusion criteria, and methods to be used in this scoping review as outlined on the Open Science Framework website (https://osf.io/by2qd/, accessed on 8 February 2025).

### 2.2. Review Question

We utilized the PEO framework [21] to develop our research question. In this framework, the Population (P) refers to the population living in Nepal, the Exposure (E) pertains to contaminated drinking and irrigation water, and the Outcomes (O) focus on food- and waterborne diseases. The scoping review was guided by the following overarching research question: What is the current state of research on food and waterborne diseases affecting the population in Nepal, particularly in the context of contaminated drinking and irrigation water?

### 2.3. Search Strategy

To identify subject headings and free-text terms, we conducted a preliminary search in Google Scholar and EBSCO databases, utilizing the *Choose Databases* feature to search multiple databases simultaneously. This method led us to 15 relevant studies, which we used to identify potential key terms [22,23,24,25,26,27,28,29,30,31,32,33,34,35,36]. 

Key terms were identified for each of the concepts in the PEO framework, listing the population living in Nepal and its regions (P), various sources for drinking and irrigation water (E), and specific health outcomes related to food- and waterborne diseases (O). Refer to Table 1 in the protocol for all search terms we identified to develop the search strategy (https://osf.io/by2qd/, accessed on 8 February 2025). The terms within each concept were combined using the Boolean operator OR, and all concepts were combined with the Boolean operator AND. The terms for the Exposure (E) concept were combined with the proximity operator (N, NEAR, W, depending on database syntax) to reflect all combinations of phrases where these terms were used (e.g., “water contamination” vs. “contamination of water”). Double quotation marks were used to search as an exact phrase (e.g., “ground water”), and truncation was employed to find any variations of the stem word (e.g., pollut* will find *pollution*, *pollutants, polluting*, *polluted*, etc.). 

Our public health librarian (NT) developed a single-line general search strategy to ensure reproducibility, transparency, and time efficiency [37]. The search strategy was adapted to the syntax and commands for each database. Additionally, the search string did not include terms related to health outcomes, as this could have reduced search recall and risked excluding potentially relevant studies [38]. For example, studies with health outcomes other than those listed in Table 1 of the protocol, such as “worm infestation,” “arsenicosis,” “Typhoid fever,” “Giardia lamblia,” and “Enteric fever,” would have been overlooked (https://osf.io/by2qd/, accessed on 8 February 2025).

### 2.4. Information Sources (Databases)

We searched seven databases for literature published from database inception to 5 July 2023: Academic Search Ultimate, Agricola, GreenFILE, GeoRef, MEDLINE (all on the EBSCO platform), Scopus (Elsevier), and Web of Science (Clarivate). The final search strategies for each database are presented in Supplementary Material S1. No search has been conducted for gray literature. 

### 2.5. Eligibility Criteria

As outlined in the study protocol, Table 3 (https://osf.io/by2qd/), predefined criteria were used to identify peer-reviewed studies in academic journals that met the eligibility criteria. These criteria included studies of people living in Nepal, studies that addressed human health and water quality, studies that used any methodological design, and studies that were published in English with no restriction on the publication year (https://osf.io/by2qd/, accessed on 8 February 2025).

### 2.6. Selection of Sources of Evidence

Records from database searches were downloaded in Zotero, a citation management software, and duplicates were removed. The unique records were then transferred to Rayyan, a software collaborative platform designed for screening in systematic and systematic-like reviews. Two reviewers (SC, AC) independently screened the unique records in two phases: title/abstract and full-text screening. Any discrepancies in the decision-making process were resolved through discussion, and whenever a decision could not be made, a third reviewer (LM) was consulted. The full text was pulled for the unique studies that met the eligibility criteria during the initial title/abstract screening. These full-text studies were then reviewed and reevaluated to confirm their suitability for inclusion in the analysis based on the predefined eligibility criteria.

### 2.7. Data Charting Process 

Data from the studies that met the eligibility criteria were extracted into a predefined Excel spreadsheet by one reviewer (AC), with a second reviewer (SC) verifying the accuracy of the entered data. Based on the PRISMA-ScR guidelines, the quality of each study was not assessed as “scoping reviews do not aim to produce a critically appraised and synthesized result/answer to a particular question, [they] rather aim to provide an overview or map of the evidence” [39]. 

### 2.8. Data Items 

The following data were extracted: record identifiers (e.g., objectives, methodology, sample size, Nepal region), water usage (drinking, irrigation, spring, river, well, etc.), water source (surface, wastewater, spring, river, well, glacier, rainfall, lake, pond, groundwater, harvested rainwater, etc.), type of contamination (microbial—bacteria, virus, protozoa, algae; and chemical—pesticides, metals, fertilizers), assessment of water quality, location (urban, rural, semi-urban), health outcomes, key findings, and recommendations.

### 2.9. Synthesis of Results 

We conducted a thematic analysis of the included data, coding the information related to this scoping review’s objectives by documenting the descriptive themes and generating analytical themes. A flow diagram was developed to illustrate the process of identifying studies that met the eligibility criteria. Visualizations were generated using Microsoft Excel and Microsoft Power BI (Sankey, filled map, heatmap). Articles focusing on both microbial and chemical contaminants in water sources across multiple locations in Nepal were summarized in tabular form. Future recommendations within the included studies were categorized into six common themes and presented in a separate table. 

## 3. Results

### 3.1. Selection of Sources of Evidence 

**Figure 1:**
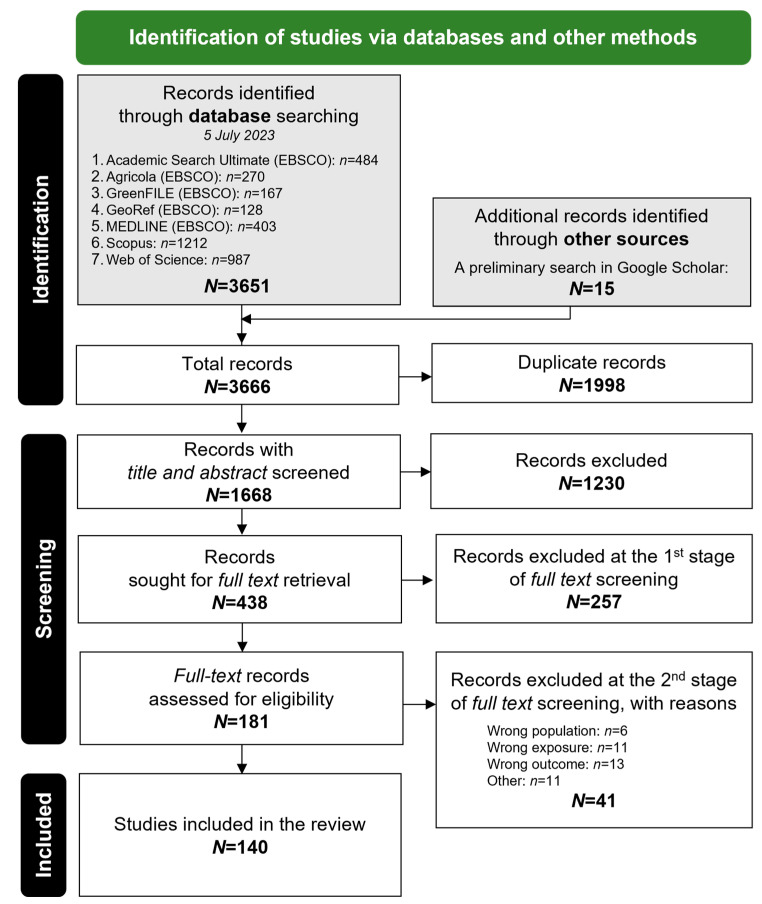
PRISMA flow chart of the study search and selection process.

The study search and selection process is illustrated in Figure 1, providing an overview of each step taken to identify and select relevant studies for inclusion.

A total of 3666 records were identified. After 1998 duplicates were removed, 1668 unique records were screened for titles and abstracts. Of these, 1230 records were excluded, and 438 were sought for full-text retrieval and reviewed in two stages. After consulting the full text for details during the first stage, we reviewed the studies initially marked as “Maybe” and “Included” in Rayyan, and 257 were excluded. During the second stage, we reassessed the remaining 181 records for eligibility, resulting in the exclusion of 41 studies [40,41,42,43,44,45,46,47,48,49,50,51,52,53,54,55,56,57,58,59,60,61,62,63,64,65,66,67,68,69,70,71,72,73,74,75,76,77,78,79,80] (Supplementary Material S2). Following the reassessment process, we identified 140 studies [22,23,25,26,27,28,29,30,33,34,35,36,81,82,83,84,85,86,87,88,89,90,91,92,93,94,95,96,97,98,99,100,101,102,103,104,105,106,107,108,109,110,111,112,113,114,115,116,117,118,119,120,121,122,123,124,125,126,127,128,129,130,131,132,133,134,135,136,137,138,139,140,141,142,143,144,145,146,147,148,149,150,151,152,153,154,155,156,157,158,159,160,161,162,163,164,165,166,167,168,169,170,171,172,173,174,175,176,177,178,179,180,181,182,183,184,185,186,187,188,189,190,191,192,193,194,195,196,197,198,199,200,201,202,203,204,205,206,207,208] that met the eligibility criteria and were subsequently included in this review (Supplementary Material S3). 

### 3.2. Characteristics of the Included Studies 

The organizational structure of this review article is based on three key categories: location (urban, rural, both, and non-specified), water sources (groundwater, surface water, and multiple other sources), and water usage (drinking, irrigation, both, and non-specified). This approach not only allowed for a more streamlined and systematic evaluation of the literature but also enabled us to address additional questions—for instance, how many studies investigated both irrigation and drinking water, or how many drew samples from multiple provinces. It is also worth noting that among microbial contaminants, some studies focused on identifying total coliforms, fecal coliforms, and/or *Escherichia coli* (*E. coli*). *E. coli* is a subset of fecal coliforms, which in turn are a subset of total coliforms. To maintain consistency with the original literature, the terminology used in each original study has been preserved without modification. 

**Figure 2:**
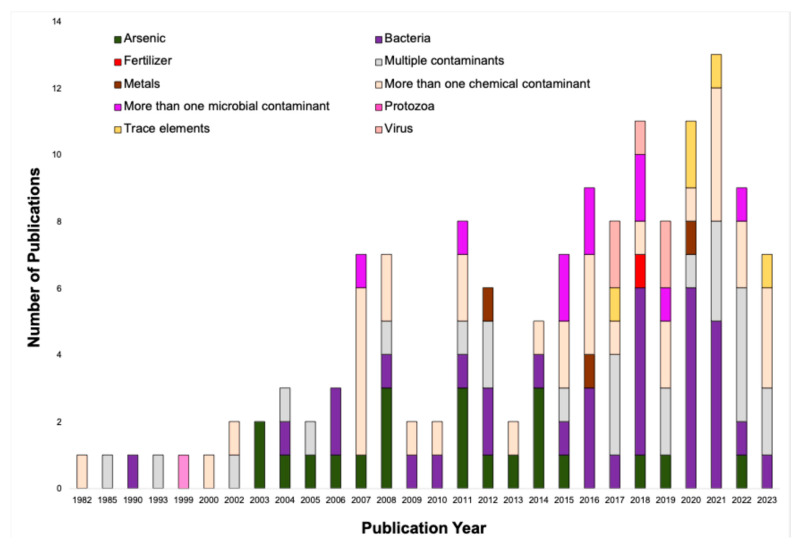
Trends in the number of publications focusing on microbial and chemical contaminants in irrigation and drinking water in Nepal across the study period 1982–2023.

Between 1982 and 2023, a total of 140 articles were published that met our screening criteria, with the highest number appearing in 2021 (13 articles), followed by 2018 and 2020 (both with 11 articles) (Figure 2). Publication rates increased significantly over the last decade (2013–2023) compared to earlier years. Out of the 140 records analyzed, 111 studies utilized quantitative research methodologies, 24 employed mixed methods (analyzing both quantitative and qualitative data), and five relied on qualitative approaches [209]. In 2021, 12 studies employed quantitative methods, and one study employed mixed methods. In 2018, nine studies employed quantitative methods, and two studies employed mixed methods. In contrast, in 2020, seven studies utilized quantitative methods, and four studies employed mixed methods. The earliest study included in the scoping review was a quantitative study published in 1982 [199]. The characteristics of the included studies are summarized in Supplementary Material S4. 

Of the 140 studies included, 95 focused exclusively on either microbial (40 studies, 28.5%), chemical (30 studies, 21.4%), or both types of contaminants (25 studies, 17.9%), regardless of the methodology used. Additionally, multiple chemical contaminants were assessed in 35 studies (25%), while multiple microbial contaminants were reported in 10 studies (7%).

#### 3.2.1. Overview of Studies 

##### Location

**Figure 3:**
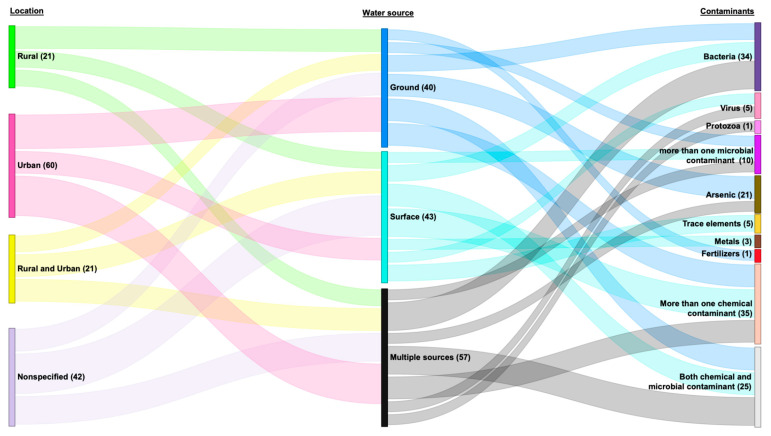
Sankey plot illustrating locations across Nepal (far-left bars and text), different *water sources* studied within those locations (middle bars and text), and identified microbial and chemical contaminants (far-right bars and text). Numbers in parentheses after the text indicate the number of studies reporting each category. Color bands indicate (1) the locations where *water sources* were studied and (2) the contaminants associated with these water sources.

Regarding the study locations, out of the 140 studies included, 21 (15%) were conducted exclusively in rural areas, 60 (42.86%) exclusively in urban areas, and 17 (12.14%) were conducted in both rural and urban locations (Figure 3). Furthermore, 42 (30.0%) studies reported only on the province from which the water samples were collected, without specifying whether the samples were from urban or rural areas.

##### Water Sources

Forty studies (28.57%) focused solely on testing groundwater, while 43 studies (30.71%) exclusively examined surface waters (Figure 3). Additionally, 57 studies (40.71%) tested both ground and surface water, as well as multiple other water sources, including bottled water, wastewater, jar water, tap water, dhunge dharas, and spout water. For this review, water sources such as seesaw wells, sunken wells, deep/shallow tube wells, hand-pump wells, dug wells, stone spouts, and bore wells are categorized as groundwater sources. While springs, streams, rivers, lakes, glaciers, and ponds are classified as surface water sources. 

Across studies that evaluated groundwater sources, arsenic was identified as the major contaminant (*n* = 20; 50%), followed by bacterial contamination (*n* = 6; 15%). Compared to studies evaluating groundwater and other sources, those focusing solely on surface water reported trace elements (*n* = 5; 11.63%) and metals (*n* = 3; 6.98%). Most studies testing surface waters (*n* = 25; 58.14%) identified chemical contaminants, including ions, trace elements, nitrogen, nitrates, chloride, manganese, and sulfur (Figure 3). Overall, Figure 3 highlights that both geography and water source type strongly influence patterns of water contamination.

##### Water Usage 

**Figure 4:**
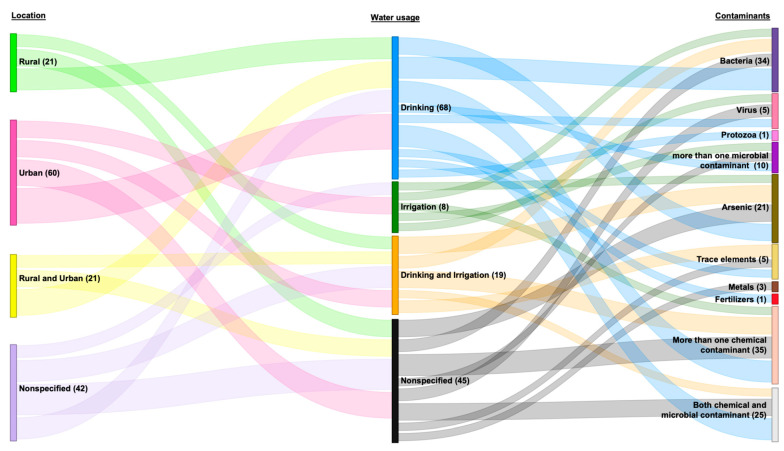
Sankey plot illustrating locations across Nepal (far-left bars and text), different *water usage* studied within those locations (middle bars and text), and identified microbial and chemical contaminants (far-right bars and text). Numbers in parentheses after the text indicate the number of studies reporting each category. Color bands indicate (1) the locations where *water usage* was studied and (2) the contaminants associated with these water sources.

In the context of water usage, the 140 included studies primarily focused on drinking water sources, with 68 studies (48.57%) exclusively examining them. Eight studies (5.71%) focused solely on irrigation water sources, while 19 (13.57%) investigated *both drinking and irrigation* water sources. Moreover, 45 studies (32.14%) did not specify the intended use of the tested water (Figure 4).

Among the microbial contaminants found in drinking water, bacteria were the most prevalent in most studies (*n* = 23; 33.82%). Notably, only one study reported the presence of protozoa [175], while two studies identified viruses [142,195]. Regarding chemical contaminants, most studies commonly reported arsenic (*n* = 9; 13.24%). Additionally, trace elements and fertilizers were detected in two studies (2.94%) [157,169] and one study (1.47%) [113] respectively (Figure 4). Eighteen articles studied more than one contaminant in their drinking water samples [22,27,30,82,83,87,95,110,146,147,160,165,166,188,190,192,197,203] (Table 1). 

In the studies that focused on irrigation waters, arsenic (*n* = 2, 20%) [98,99], bacteria (*n* = 1) [34] and viruses (*n* = 1) [185] were reported as the major contaminants. Of the 45 studies that did not specify the use of water that was being tested, three (6.67%) identified metal contamination [174,191,198] (Figure 4).

#### 3.2.2. Overview of Contaminants in Studies by Province

**Figure 5:**
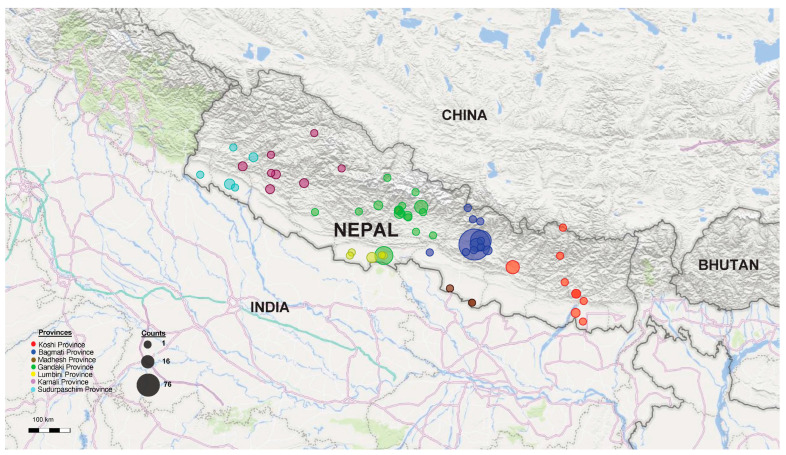
A map representing the number of studies identified from the seven provinces of Nepal. The colors represent the seven different provinces, and the circle size represents the number of studies evaluating water samples from a location in each province.

For subsequent analysis, we classified the 140 studies by provinces (Bagmati, Gandaki, Karnali, Koshi, Lumbini, Madhesh, Sudurpaschim), multiple provinces, and non-specified provinces. Among these 140 studies, 110 (78.57%) focused on a single province, with the breakdown as follows: Bagmati (*n* = 76, 69.09%), Gandaki (*n* = 15, 13.64%), Karnali (*n* = 1, 0.91%), Koshi (*n* = 12, 8.57%), Lumbini (*n* = 3, 2.73%), Madhesh (*n* = 1, 0.91%), Sudurpaschim (*n* = 2, 1.82%) (Figure 5).

**Table 1:** Articles focusing on *both microbial and chemical* contaminants in water sources across multiple locations in Nepal.

Authors	Location	Water Usage	Multiple Contaminants
Aryal, 2022 [83]	Non specified	Drinking	*E. coli*, coliforms, metals, trace elements
Aryal et al., 2012 [82]	Urban and rural		Arsenic and total coliforms
Bhandari et al., 2021 [87]	Urban		Chloride, copper, nitrate, sulfate, *E. coli*, *Citrobacter* spp., *Klebsiella* spp., *Proteus* spp., *Enterobacter* spp., *Salmonella* spp., *Shigella* spp., and *Pseudomonas*
Bittner et al., 2002 [95]	Urban and rural		Arsenic and total coliforms
Burlakoti et al., 2020 [22]	Urban		Fluoride, ammonia, *E. coli*, and total coliforms
Guragai et al., 2017 [110]	Urban		Iron, manganese, ammonia, chlorine, and *E. coli*
Merz et al., 2004 [146]	Non specified		Fecal coliform, phosphate, nitrate
Moravek et al., 2019 [147]	Non specified		Total coliforms, *E. coli*, *Giardia*, *Salmonella*, *Shigella*, nitrate, ammonia, and total phosphorus
Pant 2011 [27]	Urban		Chloride, iron, arsenic, fluoride, and total coliforms
Pradhan et al., 2005 [165]	Rural		*E. coli*, total coliforms, pH, iron, chlorine, total hardness, chloride, nitrogen, ammonia, phosphate, phosphorus, and fluoride.
Poudel & Basi-Chipalu 2022 [160]	Urban		Chloride, nitrate, iron, total coliforms, *Salmonella*, *Shigella,* and *Vibrio*
Pradhan et al., 2022 [166]	Non specified		Ammonia, nitrate, chloride, fluoride, calcium, *E. coli,* and total coliforms
Sarkar et al., 2022 [30]	Urban		Total and fecal coliforms, aluminum, arsenic, barium, beryllium, boron, cadmium, cobalt, chromium, copper, fluoride, iron, mercury, manganese, molybdenum, nickel, lead, antimony, selenium, thallium, uranium, vanadium, and zinc
Subedi et al., 2017 [190]	Urban and rural		Fecal coliforms, ammonia, nitrate, chloride, and nitrite
Subedi et al., 2012 [188]	Urban		Nitrate, calcium, iron, and fecal coliforms
Tamrakar et al., 2017 [192]	Urban		Calcium, magnesium, iron, manganese, total ammonia, sodium, potassium, fluoride, arsenic, aluminum, total chlorides, total chlorine, and fecal coliforms
Thapa et al., 2019 [197]	Urban		Calcium, magnesium, sodium, potassium, and total coliforms
Warner et al., 2008 [203]	Urban		Total coliforms, *E. coli*, nitrate, ammonia, heavy metals, arsenic, mercury, iron, sulphate, phosphate, and manganese
Ghimire 1985 [106]	Urban	Non specified	Calcium, magnesium, iron, manganese, silica, chloride, phosphate, nitrogen, ammonia, coliforms, and *E. coli*
Pantha et al., 2022 [28]	Non specified		Fecal coliforms, ammonia, nitrate, and calcium
Khadka 1993 [126]	Urban		*E. coli*, total coliforms, ammonia, orthophosphate, chloride, iron, and manganese
Khadka et al., 2015 [128]	Urban		*E. coli*, phosphate, chloride, and sulphate
Poudel et al., 2021 [164]	Urban and rural		*E. coli*, total coliforms, ammonia, phosphate, chloride, iron, and nitrate
Ghimire et al., 2023 [108]	Urban	Both drinking and irrigation	Sodium, potassium, calcium, magnesium, chloride, bicarbonate, nitrate, ammonium, iron, phosphate, sulphate, and total coliforms
Ghimire et al., 2023 [109]	Urban		Sodium, potassium, calcium, magnesium, ammonium, phosphate, sulphate, chloride, bicarbonate, nitrate, iron, and total coliforms

Twenty-three (16.43%) out of the 140 included studies covered more than one province: Koshi, Madhesh, Lumbini, and Sudurpaschim (*n* = 1, 4.35%) [208]; Bagmati, Madhesh, Gandaki, and Lumbini (*n* = 1, 4.35%) [135]; Bagmati, Lumbini, and Gandaki (*n* = 1, 4.35%) [102]; Gandaki and Bagmati (*n* = 3, 13.04%) [106,118,169]; Karnali and Sudurpaschim (*n* = 2, 8.70%) [23,86]; Koshi, Bagmati, and Gandaki (*n* = 2, 8.70%) [93,174]; Koshi, Bagmati, and Karnali (*n* = 1, 4.35%) [167]; Lumbini and Gandaki (*n* = 9, 43.48%) [81,98,99,134,137,148,149,206,207]; Gandaki and Koshi (*n* = 1, 4.35%) [198], Madhesh, Lumbini, and Gandaki (*n* = 1, 4.35%) [180] and Bagmati, Madhesh, Koshi, Lumbini, and Sudurpaschim (*n* = 1, 4.35%) [36]. 

Seven publications (5.00%) did not specify the province from which the study samples were collected [29,146,176,179,200,201,202].

##### Bagmati Province

Out of the 76 studies focusing on water samples from the Bagmati region, the majority (*n* = 56, 73.68%) examined water samples from urban areas. Among these, 48 (85.71%) utilized quantitative methods, while eight (14.29%) employed mixed methods to analyze their data. In contrast, three studies (3.98%) focused on rural locations, all using mixed methods. Additionally, seven studies (9.21%) investigated both urban and rural locations, with one (14.29%) using mixed methods and six (85.71%) employing quantitative methods. Lastly, ten studies (13.16%) did not specify the study location, and all utilized quantitative methods. 

Of the 76 studies that evaluated water from the Bagmati province, 41 identified **health outcomes**, with the predominant ones being defined as waterborne illnesses, including gastroenteritis, diarrhea, and dysentery, resulting from microbial contamination. The other major health outcomes or health risks were related to chemical or inorganic contaminants in the water sources. They were listed as arsenicosis, high hazard and cancer index, thyroid diseases, neural tube defects, and hypertension.

*Studies focusing on exclusively urban locations*

Among the studies focusing on urban locations, 15 examined water from multiple groundwater sources, including shallow wells, deep tube wells, and handpump wells [26,27,97,107,109,113,126,129,140,141,159,172,178,181,193]. Additionally, ten studies examined surface water sites, with nine out of ten focusing on the Bagmati River [88,89,91,92,122,128,155,162,194], and one on the Kodku River [107]. Furthermore, 31 studies did not specify the water source being tested. 

*Contaminants found in both drinking and irrigation water samples in studies focusing exclusively on urban locations*

Regardless of the water source, 29 studies analyzed **samples of drinking water,** of which 15 studies reported on microbes, including bacteria, viruses, and protozoa [25,33,85,103,104,139,140,141,142,143,175,181,183,189,195], four reported on chemical contaminants [97,113,129,172] and ten studies reported on *both microbial and chemical* contaminants [22,27,30,44,110,160,188,192,197,203]. Additionally, four articles reported on microbes, including waterborne protozoa, bacteria, and viruses in **irrigation water samples** [34,182,184,185]. Eight tested ***both drinking and irrigation* waters** and identified more than one chemical contaminant [89,91,92,107,155] or only bacteria, including *Acinetobacter*, *Pseudomonas*, *Flavobacterium*, and *Arcobacter* [178] and both microbes and chemical contaminants [108,109]. 

Five studies exclusively identified various **chemical contaminants** in the shallow tube and dug wells, including nitrogenous fertilizers [113], nitrogen, iron, and chloride [159,172], and multiple chemical contaminants like ammonia, nitrate, mercury, iron, magnesium, chloride, and sulfate [97,129]. Six studies exclusively identified bacteria and protozoa among the **microbial contaminants** [26,140,141,181,183,193]. In studies that identified bacterial contaminants, two studies highlighted *Bacteroidales* [140,141], two identified *E. coli* [181,183], one identified *Acinetobacter* [193], and one identified *Arthrobacter* and *Legionella* [26]. One study detected two protozoa, *Giardia* and *Cryptosporidium* [181]. Among the articles that tested for ***both microbial and chemical* contaminants**, 10 articles identified several bacteria from the *Enterobacteriaceae* family, along with metals and trace elements (Table 1). 

Three studies focused on bottled water samples [22,103,104], two of which detected microbial contaminants [103,104], while one study [22] also identified chemical contaminants such as fluoride and ammonia. A single study [142] focused on tanker water and identified bacterial (*E. coli*) and viruses (enteroviruses, noroviruses of genogroup II (NoVs-GII), human adenoviruses (HAdVs), and group A rotaviruses). Three studies assessed jar water [139,189,195], revealing contamination with bacteria (*E. coli* and total coliforms), viruses (group A rotaviruses, enteroviruses, adenoviruses, and noroviruses of genogroup I), and protozoa (*Cryptosporidium* and *Giardia*). Moreover, one study tested tap water and detected *Aeromonas* spp. in the samples [163], while another study [130] examined wastewater samples and identified heavy metals (e.g., iron, lead, zinc, and arsenic).

Out of the eight studies examining urban water sources for *both drinking and irrigation*, five focused on surface water sources from the Bagmati River [89,90,92,155] and the Kodku River [107]. Two studies evaluated groundwater from dug wells [108,109], while one study tested multiple water sources, including shallow dug wells, deep tube wells, and rivers [178]. While contaminants like arsenic and trace elements were identified in all surface water sources, both inorganic (iron, calcium) and microbial (total coliform) contaminants were identified in dug well samples [108,109]. One study examined microbial contaminants and identified *Acinetobacter*, *Pseudomonas*, *Flavobacterium*, and *Arcobacter* [178] from multiple water sources, such as dug wells, deep tube wells, and rivers.

*Contaminants found in water samples that did not specify water usage in studies focusing exclusively on urban locations*

Fifteen studies assessing urban water sources **did not specify the intended use of the water,** but identified multiple chemical and microbial contaminants [26,88,114,115,116,121,126,128,130,159,162,163,187,193,194]. Four studies focused **exclusively on groundwater sources** identified different types of contaminants—two studies identified only bacteria (*E. coli*, total coliforms, *Acinetobacter*, *Neisseria*, *Streptococcus* and *Propionibacterium*) [26,193], one study identified only inorganic contaminants like nitrate and iron [159], and one study identified *both microbial and chemical* contaminants [126]. 

Five additional studies exclusively evaluated water samples from **surface water sources** such as rivers [88,121,128,162,194]. Tandukar et al. [194] identified enteric viruses (enteroviruses and saliviruses), protozoa (*Cryptosporidium* and *Giardia*), and bacteria (total coliforms, *E. coli*, and *Enterococcus* spp.), while Khadka et al. [128] also identified heavy metal contaminants in the Bagmati River. Other contaminants, including heavy metals and trace elements, were also identified in three studies [88,121,162].

Six studies evaluated **multiple water sources**, including shallow dug wells, tube wells, sewage pipes, piped water (surface water that is being transported through pipes into homes and public tap/standpipes [210]), jar water, tanker water, and rivers [114,115,116,130,163,187]. Three studies investigated bacterial contaminants, with two focusing on *E. coli* [116,187], and one on *Aeromonas* [163]. Among the *E. coli* studies, one specifically examined the presence of Shiga toxin-producing strains [187].

*Studies focusing exclusively on rural locations*

Among the studies focusing on rural areas, three specifically examined drinking water samples from different sources: piped water, tube wells, and open wells [35]; streams [100]; and stone spouts, ponds, streams, and wells [165]. Each of the three studies employed mixed methods in their research. All of the studies identified fecal coliforms [35,100,165]. Additionally, Pradhan et al. [165] also identified iron, chlorine, chloride, nitrogen, ammonia, phosphate-phosphorus, and fluoride in the tested water sources, while Dahal et al. [100] also identified ammonia, nitrate, orthophosphate, lead, copper, zinc, sodium, and potassium. 

*Studies focusing on both urban and rural locations*

Two additional studies focused on drinking water samples from urban and rural areas [95,190]. Both studies drew on multiple sources, including groundwater and surface water samples. While Subedi et al. [190] identified only microbial contaminants, both inorganic (arsenic) and microbial contaminants (*E. coli* and other bacteria) were identified by Bittner et al. [95]. Five studies evaluated water samples from both urban and rural locations without specifying the intended use of the water collected [120,122,123,164,168]. Among the five studies mentioned above, only one study [123] focused on both ground and surface waters, while all the other studies concentrated solely on surface water samples. Three studies identified inorganic contaminants [120,122,168], including metals like lead [168]. One study identified microbial contaminants (*E. coli*) [164]. 

Five studies evaluated drinking water quality without specifying whether the samples were collected from urban or rural locations. Among these studies, two evaluated groundwater [166,196], and three tested multiple water sources like stone spouts, rivers, snowmelt, and rainwater [124,147,186]. The referenced studies detected multiple bacteria, including *E. coli*, total coliforms, *Salmonella*, *Shigella*, *S. typhi*, and *S. paratyphi*, as well as protozoa such as *Giardia*. Additionally, one study reported the presence of chemical contaminants, including nitrate and ammonia [166]. Two studies, which did not specify whether the samples were collected from urban or rural locations, evaluated *both drinking and irrigation* water sources from surface waters such as rivers [158,199]. While the study by Upadhyaya & Roy [199] detected metal contaminants, the study by Pantha et al. [158] identified bacterial contaminants (*Clostridium*, *Prevotella*, *Arcobacter*, *Lactobacillus*, *Enterococcus*, and *Streptococcus*). Furthermore, three studies [90,94,170] evaluated surface waters from rivers without specifying the location of the collected samples or the intended use of the water source. These studies reported both chemical contaminants [90,170] and microbial contaminants (bacteriophages) [94]. 

##### Gandaki Province

Of the 15 studies from the Gandaki Province, one (6.67%) focused on urban areas, two (13.34%) focused on rural areas, and five (33.34%) investigated both urban and rural locations. In contrast, seven (46.67%) did not specify the sampled regions. 

The **sole urban study** within this province, a quantitative study [144], examined heavy metal contamination, particularly lead, in Phewa Lake without specifying the water’s usage. 

In the **rural location**, one of the two studies, a mixed-methods study by Maharajan et al. [136], analyzed arsenic levels in groundwater used for drinking purposes. The other study, by Khadka and Ramanathan [127], investigated multiple chemicals in lakes within the Pokhara Valley without specifying water usage.

Of the five studies examining **both urban and rural locations**, three quantitative studies focused exclusively on drinking water from various sources, such as springs, borewells, rivers, and reservoir tanks, and surface sources within the Besishahar municipality [83], Mygadi district [82] and Lakes Begnas and Rupa [157]. One study exclusively focused on trace elements [157], while the other two investigated microbial and chemical contaminants [82,83]. The remaining two studies, conducted in urban and rural areas, examined both irrigation and drinking water, with a strong focus on chemical contaminants. Pant et al. [153] examined trace elements in the Badigad river basin, while Yadav et al. [204] focused on arsenic levels in the Terai region.

In **unspecified locations within the province**, three out of seven studies examined both irrigation and drinking water sources. These three studies focused exclusively on chemical or inorganic contaminants, including arsenic [205], trace elements [157], and multiple chemical contaminants [156]. Yadav et al. [205] focused on groundwater sources in the Nawalparasi district, while Pant et al. examined surface water sources from the Seti River Basin [154] and the Gandaki River Basin [157]. The other four studies from Gandaki Province exclusively investigated surface water sources without specifying the water usage [28,96,105,154]. Of these four studies, three focused on chemical contaminants [96,105,154], and one study examined *both microbial and chemical* contaminants in the Tanahun district’s springs in the western mid-hill region of Nepal [28]. 

Six studies from the Gandaki Province also reported on multiple **health outcomes** or health risks: high cancer index measurements [153,154,157], arsenicosis, skin lesions [136,204], and gastrointestinal problems due to drinking water with increased levels of sulfates [205].

##### Karnali Province

A mixed-method study conducted in the rural Karnali Province examined drinking water sourced from communal taps or piped water sources [145]. The study found *E. coli* in the tested water sources, and diarrhea was reported as the associated health outcome.

##### Koshi Province

Among the 12 studies from Koshi Province, four (33.34%) focused on rural locations, whereas eight (66.67%) did not specify the region. Of the four studies focusing on **rural locations**, only one [138] investigated metal contamination (e.g., manganese, iron, and arsenic) in groundwater sources (borewells and tube wells) used for drinking. Within the **unspecified locations**, four studies explored drinking water quality [125,131,150,151]. They identified microbial contaminants (e.g., *Streptococcus* and fecal coliforms) in bottled water and municipal taps [151], streams, and household storage containers [125], and various sources [131,150].

Out of the seven studies that **did not specify water use**, three focused on microbial contaminants, specifically bacteria, in rural locations. The remaining four examined various chemical contaminants in surface waters from unspecified locations. The researchers [132,133] primarily investigated culturable bacteria (e.g., *Acinetobacter*, *Aeromonas*, *Bacillus*, *Sanguibacter*, etc.) in surface, glacier melt waters, and lakes in the Mount Everest region. Shreshtha and Shakya [177] identified *Vibrio* species in multiple water sources (rivers, ponds, taps, and sewage) within Sunsari, Terai region. The identified chemical or inorganic contaminants included trace elements [161] and mercury [191] from the Koshi River Basin; unspecific metals from Gokyo Valley, Everest National Park, Nepal [173]; and persistent organic pollutants (POPs) and polycyclic aromatic hydrocarbons (PAHs) from Sagarmatha National Park, Solu-Khumbu District, in northeastern Nepal [111].

Only two studies reported **health outcomes**: one focused on diarrhea caused by vibrios [177], and the other examined the high risks of lead and cadmium pollution [174].

##### Lumbini Province

Of the three quantitative studies reviewed, one [84] was conducted in rural locations, while the other two [112,171] did not specify their location. Atreya et al. [84] investigated microbial contaminants, specifically total coliforms, in drinking water sourced from tube wells. Sapkota et al. [171] and Gyawali et al. [112] focused on chemical or inorganic contaminants, including multiple chemicals (calcium, magnesium, sodium, iron, and nitrogen compounds) and arsenic. Sapkota et al. [171] researched multiple chemical contaminants in surface waters used for drinking and irrigation, while Gyawali et al. [112] analyzed groundwater without specifying the intended use of the water samples. The primary health outcomes reported in the two studies were waterborne illnesses and health issues associated with elevated arsenic levels.

##### Madhesh Province

In Gaur Municipality, located in the Rautahat district within the Madhesh Province, a quantitative study [119] examined arsenic contamination in groundwater. The study did not report any health outcomes or specify the purposes of water usage.

##### Sudurpaschim Province

In Sudurpaschim Province, two studies assessed surface water, focusing on physico-chemical parameters. One study conducted in a rural area [101] and another in an unspecified location [117] explored these parameters in depth. Dumaru et al. [101] conducted a comprehensive evaluation of spring water quality in the Thuligaad watershed. This watershed spans the Kailali and Doti districts, serving multiple purposes, including drinking water and irrigation. Their study employed a mixed-methods approach, combining qualitative and quantitative analyses to provide a holistic understanding of the water quality in this region. On the other hand, Joshi and Devkota [117] focused on the Ghodaghodi lakes in Western Terai, Nepal. Unlike the study by Dumaru et al. [101], this research did not specify the intended use of the water samples. 

Neither of these studies addressed health outcomes associated with the water quality under examination.

##### Multiple Provinces

Among the 23 studies encompassing multiple (2–5) provinces in Nepal, 12 focused on drinking water samples: Lumbini and Gandaki [81,134,137]; Kamali and Sudurpaschim [23,86]; Gandaki and Bagmati [169]; Bagmati, Gandaki, and Lumbini [102]; Koshi, Bagmati, and Kamali [167]; Koshi, Bagmati, and Gandaki [93]; Bagmati, Madhesh, and Lumbini [135]; Madhesh, Lumbini, and Gandaki [180]; and Bagmati, Koshi, Lumbini, Madhesh, and Sudurpaschim [36]. Of these studies, four employed mixed-methods [23,86,102,137], five used quantitative methods [81,93,134,167,169], and three used qualitative methods [36,135,179]. 

Six studies [36,81,134,135,137,179] examined groundwater quality from tube wells and detected contaminants such as arsenic. In contrast, one study [169] investigated surface water sources like lakes, and another [93] focused on tap water. Four studies [23,86,102,167] examined multiple water sources, including wells, piped water, and natural springs. These studies identified both chemical contaminants, such as trace elements [169] and microbial contaminants like *E.coli* [167].

Three studies, spanning multiple provinces and employing quantitative methods, examined water intended for irrigation [98,99,118]. One of these studies assessed surface water sources such as lakes [118]; the other two investigated groundwater quality from tube wells [98,99] and seesaw wells [98]. The identified contaminants included metals [118] and arsenic [98,99].

Two studies examined *both drinking and irrigation* waters sourced from groundwater, particularly tube wells, with arsenic being identified as the primary chemical contaminant [149,208]. Six studies spanning two provinces assessed water sources without specifying their intended use [106,148,174,198,206,207]. Among these studies, three focused on groundwater from tube wells, detecting arsenic contamination [148,206,207]. The remaining three studies [106,174,198] identified metal contamination in river water sources, along with microbial contamination (*E. coli* and total coliforms) [106].

Only 13 of the 22 studies examined **health outcomes**, with arsenicosis reported in seven [81,134,137,148,149,205,208]. Three studies focused on waterborne diseases such as non-specific diarrheal diseases and enteric typhoid fever [23,93,167].

##### Unspecified Provinces

Among the seven studies that did not specify the province, two [176,201] were conducted in rural areas, one [29] in both urban and rural locations, and four [146,180,200,202] in an unspecified location. These studies employed quantitative [29,200,201,202] and mixed-methods [146,176,180] research methods.

Three studies [29,176,201] focused on microbial contaminants, specifically *E. coli*, in various drinking water sources. Two studies [180,200] identified arsenic contamination in drinking water from groundwater sources, and one study [146] examined *both microbial and chemical* quality of drinking water from various sources such as natural springs (mool), improved springs or spring boxes (kuwa), stone spouts (dhunge dhara), taps (dharo; water supply through a pipe with a proper tap or open PVC pipe end), dug wells (inar), and streamlets (kholsa).

Additionally, one study that did not specify the use of water samples in the study evaluated multiple metals (arsenic, iron, manganese, and sulfur) in groundwater [202]. The reported health outcomes included chronic exposure to arsenic [180,200] and diarrheal diseases [146,201].

#### 3.2.3. Synthesis of Results

**Figure 6:**
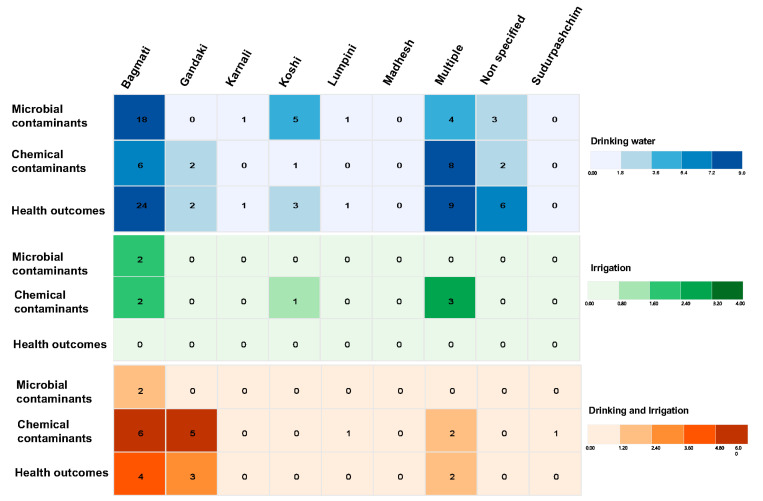
Heatmap representing the number of studies identifying microbial and chemical contaminants in water samples collected from the seven provinces and their potential related health outcomes. Darker colors represent a higher number of studies, while lighter shades represent a lower number of studies.

To provide a comprehensive understanding of the geographical distribution and impact of water contamination in the included studies, we present an analysis illustrated in Figure 6. This figure features a heatmap that visually represents the number of studies identifying microbial and chemical (or inorganic) contaminants in water samples collected from seven provinces in Nepal. The heatmap also correlates these findings with various health outcomes reported in the included studies. This visual representation facilitates the identification of contamination hotspots and understanding of the corresponding health implications across different provinces.

Among the 140 studies included in this scoping review, 32 focused on microbial contamination in **drinking water**, identifying bacteria, viruses, and protozoa. Additionally, 19 studies highlighted chemical or inorganic contamination, reporting on substances such as arsenic, fertilizers, and trace elements. In evaluations of drinking water samples, 46 studies documented various health outcomes, including conditions such as arsenicosis and waterborne illnesses (defined as diarrhea, typhoid, and enteric fever). Only two studies identified microbial contamination in assessments of **irrigation water** sources, while six studies detected multiple types of chemical contamination. 

Across studies evaluating ***both drinking and irrigation* water samples**, two studies identified bacterial contaminants, while 15 detected chemical or inorganic contaminants ranging from arsenic to trace elements. Furthermore, nine studies discussed potential health outcomes or risks, including higher cancer risks and waterborne illnesses, such as gastroenteritis.

#### 3.2.4. Recommendations Proposed by the Included Studies

Out of the 140 studies reviewed, 91 provided a range of recommendations aimed at addressing the water quality challenges in Nepal. These recommendations encompass a variety of measures essential for mitigating contamination and improving overall water safety. The proposed strategies include treatment techniques, infrastructure improvements, monitoring schemes, educational campaigns, policy reforms, and continued research (Table 2).

Overall, multiple articles highlighted the persistent contamination of water sources, particularly by arsenic, bacteria, and chemicals, which pose significant health risks to the population. To address this issue, Ahmad et al. [81] recommended early preventive measures, case management, and the supply of arsenic-safe water, while M. Aryal [83] emphasized the importance of implementing appropriate treatment methods and testing procedures. Furthermore, Aryal et al. [82] mentioned the importance of protecting natural water sources and implementing effective treatment and distribution management strategies to ensure a safe drinking water supply. Similarly, Bittner et al. [95] suggested a multifaceted point-of-use treatment regime involving filtration and disinfection to combat microbial contamination. Infrastructure improvement emerges as a key recommendation across multiple studies. Dumaru et al. [101] advocated for improved water transport infrastructure, particularly in urban areas, while Khatiwada et al. [129] emphasized the need for wastewater treatment plants in industries to mitigate environmental hazards. Several authors emphasized the importance of community awareness and education programs. Baker et al. [85] highlighted the importance of infrastructure improvements in conjunction with health campaigns to control diseases such as typhoid. Similarly, Malla et al. [141] recommended increased surveillance of water industries and microbial analysis to ensure water quality. Moreover, sustainable management practices were underscored by various studies. Meierhofer et al. [145] emphasized the need for catchment-based water management plans that involve all stakeholders, while Subedi and Aryal [189] suggested treating underground water sources and raising public awareness about water quality. Despite these recommendations, challenges remain, as highlighted by several authors. Ghimire et al. [109] stressed the need for chemical and biological treatment of groundwater, and Nicholson et al. [150] emphasized continued monitoring of water sources, especially in tourist areas.

As evidenced by this overview of recommendations in the included studies, the literature on water quality management in Nepal highlights the complexity of the issue, and emphasizes the need for integrated, holistic approaches. Recommendations include infrastructure improvement, community education, sustainable management practices, and continued monitoring and surveillance. However, effectively implementing these measures requires collaboration among stakeholders, government intervention, and sustained funding to ensure the availability of clean and safe drinking water for all.

## 4. Discussion 

Our scoping review highlights research conducted across all seven provinces in Nepal: Bagmati, Gandaki, Karnali, Koshi, Lumbini, Madhesh, and Sudurpaschim. This widespread geographical coverage underscores the extensive nature of water quality issues throughout the country, providing a robust basis for our discussion of the varying contamination levels and health impacts observed across these regions.

The highest number of studies (*n* = 76) evaluated water quality in Bagmati Province, followed by Gandaki (*n* = 15) and Koshi (*n* = 12). In contrast, the provinces with the lower number of studies were Karnali (*n* = 1), Madhesh (*n* = 1), Sudurpaschim (*n* = 2), and Lumbini (*n* = 3) (Figure 5). Regardless of the province, most of the studies included in this scoping review focused exclusively on evaluating drinking water quality (*n* = 68), while a smaller number evaluated irrigation water quality (*n* = 8). 

### 4.1. Water Quality Evaluation in Nepal Provinces

**Bagmati province**, which includes Nepal’s capital, Kathmandu, spans 13 districts and is home to 6.1 million people [212]. According to the Nepal Multiple Indicator Cluster Survey (MICS) statistical WASH report, 4.4% of the rural household population and 1.8% of the urban population in the Kathmandu Valley have unimproved drinking water [1]. Our review indicates that studies from the Kathmandu Valley show widespread contamination by *E. coli* and total coliforms in the urban community. More than one chemical contaminant and fertilizer predominated in the urban Kathmandu valley. Most of the studies from this province focused on drinking water quality (*n* = 39), while four studies exclusively tested irrigation water quality. 

**Gandaki Province** reported the second-highest number of studies (*n* = 15) and is home to approximately 2.5 million people [212]. The province is home to the popular tourist destination of Pokhara, which attracts one million tourists per year [213]. It features five major lakes: Phewa, the second largest lake in Nepal and largest in the Pokhara region; Begnas; Rupa, the third largest lake in the Pokhara region [214]; Tilicho; and Damodarkunda, along with numerous rivers and streams. Among the five major lakes, we identified studies focusing on heavy metals, trace elements, and multiple chemical contaminations in Phewa, Begnas, and Rupa within the Pokhara region. A major part of Rupa Lake is surrounded by forest and agricultural fields; hence, the high levels of trace elements can be attributed to these sources. 

Nearly 4.9 million people reside in **Koshi Province**, which is renowned for one of Nepal’s major rivers, the Koshi River [212]. Trace elements, metals, and bacteria were the predominant contaminants identified in the water samples tested from this province. 

**Karnali, Lumbini, Madhesh, and Sudurpaschim provinces,** with the lowest number of studies, are home to approximately 15.6 million people [212]. While studies examining drinking water quality in Karnali and Lumbini reported bacterial contamination, those from Madhesh and Sudurpaschim focused on chemical contaminants such as arsenic. 

### 4.2. Predominant Health Concerns Identified in the Included Studies 

Our review identified arsenicosis and other waterborne diseases as the predominant public health concerns in most of Nepal’s provinces. Arsenicosis, a debilitating condition caused by prolonged exposure to arsenic-contaminated water, poses a significant public health concern. Despite being a landlocked nation, Nepal shares similar challenges with its neighboring countries regarding arsenic contamination in groundwater and its adverse health effects on populations. Arsenic contamination in drinking water sources affects approximately 3 million people in Nepal [200]. In districts like Bara, Parsa, and Nawalparasi in the Terai region, approximately 5.1% of the population has shown clinical symptoms of arsenicosis, with over 5,200 individuals exposed to arsenic levels above 50 μg/L [215]. Other affected areas, such as Rautahat and Nawalparasi, have reported arsenicosis prevalence rates of 2.5% and 2.1%, respectively, while in Siraha and Saptari, the rates range between 1% and 3%, indicating widespread health risks associated with prolonged exposure to arsenic-contaminated groundwater [135]. Additionally, much of the country’s groundwater contains naturally occurring arsenic, sometimes at concentrations exceeding 10 ppb [216]. The prevalence of arsenicosis varies across different regions of Nepal, with higher incidences reported in areas with greater arsenic contamination [36,134,135], hence underscoring the urgent need for coordinated efforts to address water quality issues and mitigate the health impacts of arsenic contamination.

Arsenicosis manifests in various forms, including skin lesions, respiratory problems, cardiovascular diseases, and even cancers, imposing a significant burden on affected individuals and healthcare systems [217]. The primary cause of arsenic contamination in groundwater is geological, as arsenic leaches naturally from bedrock into aquifers over time. However, anthropogenic activities such as mining, industrial pollution, and agricultural practices can exacerbate arsenic contamination, further compromising water quality and public health. In Nepal, rapid urbanization, industrial growth, and agricultural intensification contribute to the contamination of water sources, exacerbating the arsenicosis crisis [216]. Additionally, inadequate infrastructure and limited access to safe drinking water increase the vulnerability of marginalized communities, thereby widening disparities in arsenicosis prevalence.

Other waterborne diseases, defined by outcomes such as non-specific diarrheal illnesses, typhoid fever, and hepatitis A, continue to impose a significant burden on public health in Nepal. According to UNICEF, 3.5 million Nepalese lack access to basic water services, leading to potential waterborne diseases, particularly among children under the age of five [1]. Inadequate water and sanitation facilities are major contributors to the high incidence of waterborne diseases in Nepal [218]. The prevalence of waterborne diseases in Nepal can be attributed to various factors, including inadequate water quality monitoring, poor sanitation infrastructure, and geographical vulnerabilities. Reliance on contaminated groundwater sources exacerbates the risk of waterborne illnesses in rural areas, where access to safe drinking water is limited. Additionally, rapid urbanization and industrialization have led to pollution of surface water bodies, further compromising water quality and public health [218].

### 4.3. Research Gaps Identified and Proposed Recommendations

Despite agriculture being Nepal’s primary economic activity, engaging around 65% of the population and contributing 31.7% to the Gross Domestic Product (GDP) [219], our study revealed a significant gap in research on evaluating irrigation water quality. This knowledge gap in assessing water quality may have significant implications for public health outcomes, particularly regarding water and food-borne disease prevalence [11,82]. Poor water quality in irrigation systems can lead to crop contamination, increasing the risk of foodborne illnesses among consumers. Therefore, addressing this research gap and ensuring the quality of irrigation water is crucial for safeguarding public health and enhancing agricultural productivity in Nepal.

Addressing these public health concerns requires a comprehensive and multifaceted approach that encompasses water quality monitoring, infrastructure development, community education, and policy interventions. Collaborative efforts involving government agencies, non-governmental organizations, and international partners are crucial for implementing sustainable solutions and ensuring access to safe drinking water for all Nepalese communities. By learning from experiences in neighboring countries and prioritizing preventive measures, Nepal can mitigate the health impacts of arsenicosis and waterborne diseases, contributing to improved overall well-being and sustainable development.

As Nepal strives to achieve its Sustainable Development Goals [220], it is imperative to assess the progress made thus far and identify areas that require heightened attention. A significant research gap persists in understanding water insecurity across Nepal, especially outside the well-studied Bagmati Province. Therefore, efforts should be directed towards provinces with lower research coverage, ensuring a comprehensive evaluation of water quality contaminants in those areas.

The prevalence of arsenicosis underscores the urgent need for concerted action to address water quality issues in Nepal and its neighboring countries. Beyond being a public health concern, arsenicosis serves as a poignant reminder of the intricate connections between water quality, environmental health, and human well-being. By expanding research efforts and implementing targeted interventions, Nepal can safeguard its population against the detrimental effects of water contamination while advancing progress toward the SDGs.

It is recommended that stakeholders, including government bodies, research institutions, and non-governmental organizations, collaborate to expand the scope of water quality studies to areas beyond Bagmati Province [211,221]. Investments in monitoring and mitigation strategies should prioritize regions with limited research coverage to ensure equitable access to safe water resources for all Nepalese citizens. Through collective action and a comprehensive approach, Nepal can effectively progress toward its water quality goals and SDG 6, ultimately ensuring healthier lives and a more sustainable environment for its citizens.

### 4.4. Strengths and Limitations

Our scoping review provides a comprehensive overview of articles that have researched drinking and irrigation water quality in Nepal, highlighting key contaminants such as bacteria and arsenic, and their potential links to waterborne diseases, including typhoid and arsenicosis. A major strength of this review lies in its systematic and rigorous approach, guided by the JBI Manual for Evidence Synthesis and PRISMA-ScR guidelines, and its extensive search across seven databases, which enabled the inclusion of diverse methodological studies (quantitative, qualitative and mixed-methods) and provided valuable insights into regional disparities (e.g., majority of the included articles focused on water samples from the Bagmati region) and gaps in water quality research. The review also provides an overview of health outcomes from both drinking and irrigation water sources, identifying critical challenges such as weak regulatory enforcement, inconsistent testing protocols, and the lack of coordination between water quality monitoring and public health planning. However, the review also has limitations. Our review article did not conduct a statistical analysis of the data from the included articles, making it difficult to identify water quality trends, if any, or potential associations with health outcomes. Furthermore, the exclusion of non-English language publications may have led to the omission of relevant studies, introducing potential language bias.

## 5. Conclusions

In summary, our scoping review highlights significant disparities in water quality research across Nepal’s provinces, with Bagmati receiving the most attention. At the same time, regions like Karnali, Madhesh, and Sudurpaschim remain under-studied. The predominant contaminants identified were arsenic, trace elements, and microbial pathogens, contributing to widespread public health concerns, including arsenicosis and other waterborne diseases. Despite agriculture’s importance, the quality of irrigation water is largely overlooked, posing risks to food safety and public health. Arsenicosis and waterborne diseases remain major concerns, driven by both natural and human factors. 

Our review suggests the urgent need for expanded monitoring and interventions, and the importance of coordinated efforts to ensure equitable access to safe water nationwide. Beyond Nepal, this article contributes to the global community by offering specific methodological insights into linking water quality phenomena with food- and waterborne disease risks in both urban and rural settings, particularly in developing countries facing similar challenges. Advancing such research and interventions is critical to reducing health disparities and supporting progress toward SDG 6.

## Figures and Tables

**Table 2:** Recommendations proposed by authors of included studies and categorized under broader themes.

Theme	Recommendations	Studies
Water Treatment and Disinfection	Disinfection of drinking water sources, especially during the monsoon season Regular maintenance of water purification filters Implementation of solar water disinfection methods Utilizing chlorine or lime for water treatment before consumption Safe disposal of solid waste and wastewater	Bhandari et al., 2021 [87]; Kannel et al., 2007 [122]; Shrestha et al., 2003 [180]; Shrestha et al., 2016 [182]; Shrestha & Shakya, 2021 [177]
Infrastructure Improvement	Infrastructure improvements, especially in Kathmandu, are crucial for controlling diseases like typhoid Improving piped water supply infrastructure Enhancing water transport infrastructure for urban areas	Baker et al., 2011 [85]; Dumaru et al., 2021 [101]; Inoue et al., 2015 [26]
Water Quality Monitoring	Increased surveillance of water quality, particularly in the jar water industry Regular monitoring of groundwater and surface water quality Implementation of groundwater monitoring programs	Bhatta et al., 2007 [93]; Maharjan et al., 2006 [135]; Malla et al., 2018 [140]; Nicholson et al., 2023 [150]; Pant et al., 2021 [152]; Pant et al., 2020 [157]
Health Education and Awareness	Conducting health education programs on water hygiene, sanitation, and safe water handling Organizing awareness programs on arsenic contamination and its health effects Motivational programs regarding safe water options Training health professionals on diagnosing and managing arsenic-related health implications	Aryal et al., 2012 [82]; Maharjan et al., 2005 [137]; Shrestha et al., 2016 [182]; Warner et al., 2008 [203]
Policy and Management	Formulation of plans and policies for sustainable groundwater management Establishment of a systematic mechanism for the surveillance and monitoring of waterborne pathogens Collaboration with stakeholders for effective water resource management	Aryal et al., 2012 [82]; Bhatta et al., 2007 [93]; Khatiwada et al., 2002 [129]; Shrestha et al., 2018 [178]; Warner et al., 2008 [203]
Further Research and Studies	Conducting further research on specific topics such as temporal variations of health risks, sediment analysis, and wastewater treatment Urgent need for more comprehensive studies to ensure water quality and safety Focus on genetic analysis of pathogens and environmental impact evaluations	Bhetwal et al., 2017 [94]; Kafle et al., 2023 [118]; Sharma et al., 2021 [211]; Thakur et al., 2015 [196]; Thapa et al., 2019 [197]; Warner et al., 2008 [203]

## Data Availability

All data related to this work are presented in the manuscript and Supplementary Materials.

## Supplementary Materials

The following supporting information can be downloaded at: https://www.mdpi.com/article/10.3390/ijerph22101526/s1, Supplementary S1: Search strategies for all databases searched; Supplementary S2: List of excluded studies with reasons; Supplementary S3: List of included studies; Supplementary S4: Characteristics of included studies.
