# Supplementary material for: Microbial and Chemical Water Quality Assessments Across the Rural and Urban Areas of Nepal: A Scoping Review
Source: Int J Environ Res Public Health. 2025 Oct 5;22(10):1526. doi: 10.3390/ijerph22101526 (PMC12563189; doi:10.3390/ijerph22101526)
Supplement: Supplementary file 1 [file ijerph-22-01526-s001.zip › Supplementary S1_Search strategies_Nepal_s drinking water.pdf]

Supplementary Material S1: General Search Strategies

Main search strategy

POPULATION (P): People in Nepal
Bagmati OR Dhaulagiri OR Gandaki OR Janakpur OR Karnali OR Kathmandu OR Koshi OR Lumbini OR Madhesh OR Mahakali OR Mechi OR Melamchi OR Narayani OR Nepal OR Nepalese OR Nepali* OR Rapti OR Sagarmatha OR Seti OR Sudurpashchim

AND

EXPOSURE (E): Contaminated water
water N3 (contamination OR drink* OR groundwater OR "ground water" OR insecurity OR irrigation OR microbiology OR pollut* OR purification OR quality OR river* OR sanitation OR security OR source* OR spring* OR storage OR suppl* OR system* OR well*)

Search strategy adapted for ***EBSCO databases***

Date: July 5, 2023

Query #	Search strings and limiters	Academic Search Ultimate	Agricola	GreenFILE	GeoRef	MEDLINE
**#1: Population** (People living in Nepal)	TI (Bagmati OR Dhaulagiri OR Gandaki OR Janakpur OR Karnali OR Kathmandu OR Koshi OR Lumbini OR Madhesh OR Mahakali OR Mechi OR Melamchi OR Narayani OR Nepal OR Nepalese OR Nepali* OR Rapti OR Sagarmatha OR Seti OR Sudurpashchim) OR AB (Bagmati OR Dhaulagiri OR Gandaki OR Janakpur OR Karnali OR Kathmandu OR Koshi OR Lumbini OR Madhesh OR Mahakali OR Mechi OR Melamchi OR Narayani OR Nepal OR Nepalese OR Nepali* OR Rapti OR Sagarmatha OR Seti OR Sudurpashchim) OR SU (	20,702	5,157	1,722	6,514	16,423

Query #	Search strings and limiters	Academic Search Ultimate	Agricola	GreenFILE	GeoRef	MEDLINE
	Bagmati OR Dhaulagiri OR Gandaki OR Janakpur OR Karnali OR Kathmandu OR Koshi OR Lumbini OR Madhesh OR Mahakali OR Mechi OR Melamchi OR Narayani OR Nepal OR Nepalese OR Nepali* OR Rapti OR Sagarmatha OR Seti OR Sudurpashchim)					
**#2: Exposure** (Contaminated water)	TI (water N3 (contamination OR drink* OR groundwater OR "ground water" OR insecurity OR irrigation OR microbiology OR pollut* OR purification OR quality OR river* OR sanitation OR security OR source* OR spring* OR storage OR suppl* OR system* OR well*)) OR AB (water N3 (contamination OR drink* OR groundwater OR "ground water" OR insecurity OR irrigation OR microbiology OR pollut* OR purification OR quality OR river* OR sanitation OR security OR source* OR spring* OR storage OR suppl* OR system* OR well*)) OR SU (water N3 (contamination OR drink* OR groundwater OR "ground water" OR insecurity OR irrigation OR microbiology OR pollut* OR purification OR quality OR river* OR sanitation OR security OR source* OR spring* OR storage OR suppl* OR system* OR well*))	342,254	211,849	90,786	370,938	351,335
**#3: Combining both concepts (P and E)**	#1 AND #2<br><br>(filtered to: peer-reviewed studies published in English)	556<br><br>**484**	335<br><br>**270**	181<br><br>**167**	577<br><br>**128**	418<br><br>**403**

Search strategy adapted for ***Scopus (Elsevier)***

Date: July 5, 2023

Query #	Search strings and limiters	Scopus results
**#1: Population** (People living in Nepal)	TITLE-ABS-KEY(Bagmati OR Dhaulagiri OR Gandaki OR Janakpur OR Karnali OR Kathmandu OR Koshi OR Lumbini OR Madhesh OR Mahakali OR Mechi OR Melamchi OR Narayani OR Nepal OR Nepalese OR Nepali* OR Rapti OR Sagarmatha OR Seti OR Sudurpashchim)	39,197
**#2: Exposure** (Contaminated water)	TITLE-ABS-KEY(water W/3 (contamination OR drink* OR groundwater OR "ground water" OR insecurity OR irrigation OR microbiology OR pollut* OR purification OR quality OR river* OR sanitation OR security OR source* OR spring* OR storage OR suppl* OR system* OR well*))	1,158,121
**#3: Combining both concepts (P and E)**	#1 AND #2<br>(filtered to: article and English)	1,587<br>**1,212**

Search strategy adapted for ***Web of Science (Clarivate)***

Date: July 5, 2023

Query #	Search strings and limiters	Web of Science results
**#1: Population** (People living in Nepal)	TS=(Bagmati OR Dhaulagiri OR Gandaki OR Janakpur OR Karnali OR Kathmandu OR Koshi OR Lumbini OR Madhesh OR Mahakali OR Mechi OR Melamchi OR Narayani OR Nepal OR Nepalese OR Nepali* OR Rapti OR Sagarmatha OR Seti OR Sudurpashchim)	32,080
**#2: Exposure** (Contaminated water)	TS=(water NEAR/3 (contamination OR drink* OR groundwater OR "ground water" OR insecurity OR irrigation OR microbiology OR pollut* OR purification OR quality OR river* OR sanitation OR security OR source* OR spring* OR storage OR suppl* OR system* OR well*))	643,016
**#3: Combining both concepts (P and E)**	#1 AND #2<br>(filtered to: article, review article and English)	1,067<br>**987**
