# Supplementary material for: Microbial and Chemical Water Quality Assessments Across the Rural and Urban Areas of Nepal: A Scoping Review
Source: Int J Environ Res Public Health. 2025 Oct 5;22(10):1526. doi: 10.3390/ijerph22101526 (PMC12563189; doi:10.3390/ijerph22101526)
Supplement: Supplementary file 1 [file ijerph-22-01526-s001.zip › Supplementary S2_Excluded studies_Nepal_s drinking water.pdf]

Supplementary Material S2: Excluded studies with reasons

Reference #	**Excluded Study** (in alphabetical order by author)	**Reason for exclusion**
[40]	Acharya, K., Khanal, S., Pantha, K., Amatya, N., Davenport, R. J., & Werner, D. (2019). A comparative assessment of conventional and molecular methods, including MinION nanopore sequencing, for surveying water quality. *Scientific Reports, 9*(1), 15726. https://doi.org/10.1038/s41598-019-51997-x	**Other** (this study has water samples, but is mainly focused on the technologies for testing)
[41]	Adhikari, S., Sharma Regmi, R., Sapkota, S., Khadka, S., Patel, N., Gurung, S., Thapa, D., Bhattarai, P., Sapkota, P., Devkota, R., Ghimire, A., & Rijal, K. R. (2023). Multidrug resistance, biofilm formation and detection of bla CTX-M and bla VIM genes in E. coli and Salmonella isolates from chutney served at the street-food stalls of Bharatpur, Nepal. *Heliyon, 9*(5), e15739. https://doi.org/10.1016/j.heliyon.2023.e15739	**Wrong exposure**
[42]	Alam, A., Islam, M., Rahman, M., Ahmed, E., Islam, S., Sultana, K., & Siddique, M. (2004). Transport of toxic metals through the major river systems of Bangladesh. *Journal of the Chemical Society of Pakistan, 26*(3), 328–332.	**No water quality assessment**
[43]	Balestrini, R., Polesello, S., & Sacchi, E. (2014). Chemistry and isotopic composition of precipitation and surface waters in Khumbu valley (Nepal Himalaya): N dynamics of high elevation basins. *Science of the Total Environment, 485*, 681–692. https://doi.org/10.1016/j.scitotenv.2014.03.096	**Wrong outcome**
[44]	Bhandari, D., Tandukar, S., Sherchand, S., Thapa, P., & Shah, P. K. (2015). Cryptosporidium infection among the school children of Kathmandu Valley. *Journal of Institute of Medicine Nepal, 37*(1), 82–87. https://hdl.handle.net/2440/123848	**Wrong exposure**
[45]	Bhatt, M. P., Hartmann, J., & Acevedo, M. F. (2018). Seasonal variations of biogeochemical matter export along the Langtang-Narayani river system in central Himalaya. *Geochimica et Cosmochimica Acta, 238*, 208–234. https://doi.org/10.1016/j.gca.2018.06.033	**Wrong outcome**

Reference #	**Excluded Study** **(in alphabetical order by author)**	**Reason for exclusion**
[46]	Bhatt, M. P., Masuzawa, T., Yamamoto, M., & Gardner, K. H. (2009). Spatial variations in chemical compositions along Langtang-Narayani river system in central Nepal. *Environmental Geology, 57*(3), 557–569. https://doi.org/10.1007/s00254-008-1325-x	**Wrong outcome**
[47]	Bhatta, R., Gurung, S., Joshi, R., Tuladhar, S., Regmi, D., Kafle, B. K., Dahal, B. M., Raut, N., Kafle, K. R., Kayastha, R., Prasad, A., Tripathee, L., Paudyal, R., Guo, J., Kang, S., & Sharma, C. M. (2022). Spatio-temporal hydrochemistry of two selected Ramsar sites (Rara and Ghodaghodi) of west Nepal. *Heliyon, 8*(11), e11243. https://doi.org/10.1016/j.heliyon.2022.e11243	**Wrong outcome**
[48]	Bhattachan, B., Panta, Y. B., Tiwari, S., Magar, D. T., Sherchand, J. B., Rai, G., & Rai, S. K. (2015). Intestinal parasitic infection among school children in Chitwan district of Nepal. *Journal of Institute of Medicine Nepal, 37*(2), 79–84.	**Wrong exposure**
[49]	Bhusal, A., & Devkota, A. (2020). Environmental variables and macrophytes of lakes of the Chitwan National Park, Central Nepal. *Limnological Review, 20*(3), 135–144. https://doi.org/10.2478/limre-2020-0014	**Wrong exposure**
[50]	Bickle, M. J., Tipper, E., Galy, A., Chapman, H., & Harris, N. (2015). On discrimination between carbonate and silicate inputs to Himalayan rivers. *American Journal of Science, 315*(2), 120–166. https://doi.org/10.2475/02.2015.02	**Wrong outcome**
[51]	Chakraborti, D., Mukherjee, S. C., Pati, S., Sengupta, M. K., Rahman, M. M., Chowdhury, U. K., Lodh, D., Chanda, C. R., Chakraborti, A. K., & Basu, G. K. (2003). Arsenic groundwater contamination in Middle Ganga Plain, Bihar, India: A future danger? *Environmental Health Perspectives, 111*(9), 1194–1201. https://doi.org/10.1289/ehp.5966	**Wrong population**
[52]	Davis, J. A. (1977). Water quality standards for the Bagmati River. *Journal of Water Pollution Control Federation, 49*(2), 227. https://www.jstor.org/stable/25039247	**No water quality assessment**

Reference #	**Excluded Study** **(in alphabetical order by author)**	**Reason for exclusion**
[53]	English, N. B., Quade, J., DeCelles, P. G., & Garzione, C. N. (2000). Geologic control of Sr and major element chemistry in Himalayan Rivers, Nepal. *Geochimica et Cosmochimica Acta, 64*(15), 2549–2566. https://doi.org/10.1016/S0016-7037(00)00379-3	**Wrong outcome**
[54]	Evans, M. J., Derry, L. A., Anderson, S. P., & France-Lanord, C. (2002). Hydrothermal source of radiogenic Sr to Himalayan rivers. *Geology, 29*(9), 803–806. https://doi.org/10.1130/0091-7613(2001)029<0803:HSORST>2.0.CO;2	**Wrong outcome**
[55]	Evans, M. J., Derry, L. A., & France-Lanord, C. (2004). Geothermal fluxes of alkalinity in the Narayani river system of central Nepal. *Geochemistry, Geophysics, Geosystems, 5*(8). https://doi.org/10.1029/2004GC000719	**Wrong outcome**
[56]	Grabczak, J., & Kotarba, M. (1985). Isotopic composition of the thermal waters in the central part of the Nepal Himalayas. *Geothermics, 14*(4), 567–575. https://doi.org/10.1016/0375-6505(85)90007-0	**Wrong outcome**
[57]	Griffioen, J., Passier, H. F., & Klein, J. (2008). Comparison of selection methods to deduce natural background levels for groundwater units. *Environmental Science & Technology, 42*(13), 4863–4869. https://doi.org/10.1021/es7032586	**Wrong population**
[58]	Gupta, R., Rayamajhee, B., Sherchan, S. P., Rai, G., Mukhiya, R. K., Khanal, B., & Rai, S. K. (2020). Prevalence of intestinal parasitosis and associated risk factors among school children of Saptari district, Nepal: A cross-sectional study. *Tropical Medicine & Health, 48*, 73. https://doi.org/10.1186/s41182-020-00261-4	**Wrong exposure**
[59]	Gupta, T., & Kumari, R. (2023). Assessment of groundwater nitrate vulnerability using DRASTIC and modified DRASTIC in upper catchment of Sabarmati basin. *Environmental Earth Sciences, 82*(9). https://doi.org/10.1007/s12665-023-10880-9	**Wrong population**
[60]	Ise, T., Pokharel, B. M., Rawal, S., Shrestha, R. S., & Dhakhwa, J. R. (1996). Outbreaks of cholera in Kathmandu Valley in Nepal. *Journal of Tropical Pediatrics, 42*(5), 305–307. https://doi.org/10.1093/tropej/42.5.305	**Wrong exposure**

Reference #	Excluded Study (in alphabetical order by author)	Reason for exclusion
[61]	Jüttner, I., Sharma, S., Dahal, B. M., Ormerod, S. J., Chimonides, P. J., & Cox, E. J. (2003). Diatoms as indicators of stream quality in the Kathmandu Valley and Middle Hills of Nepal and India. *Freshwater Biology, 48*(11), 2065–2084. https://doi.org/10.1046/j.1365-2427.2003.01138.x	**Wrong outcome**
[62]	Katz, J., West, K. P., Jr, Khatry, S. K., LeClerq, S. C., Pradhan, E. K., Thapa, M. D., Ram Shrestha, S., & Taylor, H. R. (1996). Prevalence and risk factors for trachoma in Sarlahi district, Nepal. *The British Journal of Ophthalmology, 80*(12), 1037–1041. https://doi.org/10.1136/bjo.80.12.1037	**No water quality assessment**
[63]	Khanitchaidecha, W., Shakya, M., Nakano, Y., Tanaka, Y., & Kazama, F. (2012). Development of an attached growth reactor for NH 4 –N removal at a drinking water supply system in Kathmandu Valley, Nepal. *Journal of Environmental Science & Health, Part A: Toxic/Hazardous Substances & Environmental Engineering, 47*(5), 734–743. https://doi.org/10.1080/10934529.2012.660097	**No water quality assessment**
[64]	Kumar, H. R. (2013). Causes and effects of critical change in nitrate level of ground water in Koshi region of North Bihar. *Pollution Research, 32*(4), 931–932.	**Wrong population**
[65]	Lee, L. I., Chye, T. T., Karmacharya, B. M., & Govind, S. K. (2012). Blastocystis sp.: Waterborne zoonotic organism, a possibility? *Parasites & Vectors, 5*(1), 130–130. https://doi.org/10.1186/1756-3305-5-130	**Wrong outcome**
[66]	Li, M., Zhang, Q., Sun, X., Karki, K., Zeng, C., Pandey, A., Rawat, B., & Zhang, F. (2020). Heavy metals in surface sediments in the trans-Himalayan Koshi River catchment: Distribution, source identification and pollution assessment. *Chemosphere, 244*, 125410. https://doi.org/10.1016/j.chemosphere.2019.125410	**Wrong exposure**
[67]	Mainali, J., & Chang, H. (2021). Environmental and spatial factors affecting surface water quality in a Himalayan watershed, Central Nepal. *Environmental and Sustainability Indicators, 9*. https://doi.org/10.1016/j.indic.2020.100096	**Wrong outcome**

Reference #	**Excluded Study** (in alphabetical order by author)	**Reason for exclusion**
[68]	Malla-Pradhan, R., Phoungthong, K., Suwunwong, T., Joshi, T. P., & Pradhan, B. L. (2023). Microplastic pollution in lakeshore sediments: The first report on abundance and composition of Phewa Lake, Nepal. *Environmental Science and Pollution Research International, 30*(27), 70065–70075. https://doi.org/10.1007/s11356-023-27315-4	**Other** (wrong contaminant-microplastics)
[69]	Malla-Pradhan, R., Pradhan, B. L., Phoungthong, K., & Joshi, T. P. (2022). Occurrence and distribution of microplastics from Nepal's second largest lake. *Water, Air, and Soil Pollution, 233*(10). https://doi.org/10.1007/s11270-022-05896-z	**Other** (wrong contaminant-microplastics)
[70]	Malla-Pradhan, R., Suwunwong, T., Phoungthong, K., Joshi, T. P., & Pradhan, B. L. (2022). Microplastic pollution in urban Lake Phewa, Nepal: The first report on abundance and composition in surface water of lake in different seasons. *Environmental Science and Pollution Research, 29*(26), 39928–39936. https://doi.org/10.1007/s11356-021-18301-9	**Other** (wrong contaminant-microplastics)
[71]	Miftahussurur, M., Sharma, R. P., Shrestha, P. K., Suzuki, R., Uchida, T., & Yamaoka, Y. (2015). Molecular epidemiology of Helicobacter pylori infection in Nepal: Specific ancestor root. *PLoS One, 10*(7), 1–16. https://doi.org/10.1371/journal.pone.0134216	**No water quality assessment**
[72]	Pant, R. R., Zhang, F., Rehman, F. U., Wang, G., Ye, M., Zeng, C., & Tang, H. (2018). Spatiotemporal variations of hydrogeochemistry and its controlling factors in the Gandaki River Basin, Central Himalaya Nepal. *Science of the Total Environment, 622–623*, 770–782. https://doi.org/10.1016/j.scitotenv.2017.12.063	**No water quality assessment**
[73]	Pokhrel, S., Ghimire, N. P., & Rai, S. K. (2021). Seasonal variation of algal diversity with reference to water quality in Jagadishpur Reservoir, Nepal. *Limnological Review, 21*(4), 189–199. https://doi.org/10.2478/limre-2021-0018	**Wrong exposure**
[74]	Rahman, M. M., Naidu, R., & Bhattacharya, P. (2009). Arsenic contamination in groundwater in the Southeast Asia region. *Environmental Geochemistry and Health, 31*(1), 9–21. https://doi.org/10.1007/s10653-008-9233-2	**No water quality assessment**

Reference #	**Excluded Study** (in alphabetical order by author)	**Reason for exclusion**
[75]	Shaji, E., Santosh, M., Sarath, K. V., Prakash, P., Deepchand, V., & Divya, B. V. (2021). Arsenic contamination of groundwater; a global synopsis with focus on the Indian Peninsula. *Geoscience Frontiers, 12*(3). https://doi.org/10.1016/j.gsf.2020.08.015	**Wrong population**
[76]	Sherchan, J. B., Sherpa, K., Tandukar, S., Cross, J. H., Gajadhar, A., & Shrechand, J. B. (2010). Infection of Cyclospora cayetanensis in diarrhoeal children of Nepal. *Journal of Nepal Paediatric Society, 30*(1), 23–30.	**Wrong exposure**
[77]	Sherpa, A. M., Byamukama, D., Shrestha, R. R., Haberl, R., Mach, R. L., & Farnleitner, A. H. (2009). Use of faecal pollution indicators to estimate pathogen die off conditions in source separated faeces in Kathmandu Valley, Nepal. *Journal of Water & Health, 7*(1), 97–107. https://doi.org/10.2166/wh.2009.149	**Wrong exposure**
[78]	Shrestha, A., Sharma, S., Gerold, J., Erismann, S., Sagar, S., Koju, R., Schindler, C., Odermatt, P., Utzinger, J., & Cissé, G. (2017). Water quality, sanitation, and hygiene conditions in schools and households in Dolakha and Ramechhap districts, Nepal: Results from a cross-sectional survey. *International Journal of Environmental Research and Public Health, 14*(1). https://doi.org/10.3390/ijerph14010089	**Wrong outcome**
[79]	Shrestha, R. R., Haberl, R., & Laber, J. (2001). Constructed wetland technology transfer to Nepal. *Water Science and Technology, 43*(11), 345–350. https://doi.org/10.2166/wst.2001.0701	**Wrong exposure**
[80]	Verma, A., Yadav, B. K., & Singh, N. B. (2020). Hydrochemical monitoring of groundwater quality for drinking and irrigation use in Rapti Basin. *SN Applied Sciences, 2*(3). https://doi.org/10.1007/s42452-020-2267-5	**Wrong population**
