# Supplementary material for: Microbial and Chemical Water Quality Assessments Across the Rural and Urban Areas of Nepal: A Scoping Review
Source: Int J Environ Res Public Health. 2025 Oct 5;22(10):1526. doi: 10.3390/ijerph22101526 (PMC12563189; doi:10.3390/ijerph22101526)
Supplement: Supplementary file 1 [file ijerph-22-01526-s001.zip › Supplementary S3_Included studies_Nepal_s drinking water.pdf]

Supplementary Material S3: Included Studies

**Reference #**	**Included Studies** (in alphabetical order by author)
[81]	Ahmad, S. A., Maharjan, M., Watanabe, C., & Ohtsuka, R. (2004). Arsenicosis in two villages in Terai, lowland Nepal. *Environmental Sciences: An International Journal of Environmental Physiology and Toxicology*, *11*(3), 179–188.
[82]	Aryal, J., Gautam, B., & Sapkota, N. (2012). Drinking water quality assessment. *Journal of Nepal Health Research Council*, *10*(22), 192–196.
[83]	Aryal, M. (2022). An analysis of drinking water quality parameters to achieve sustainable development goals in rural and urban areas of Besisahar, Lamjung, Nepal. *World Water Policy*. https://doi.org/10.1002/wwp2.12073
[84]	Atreya, K., Panthee, S., & Sharma, P. (2006). Bacterial contamination of drinking water and the economic burden of illnesses for the Nepalese households. *International Journal of Environmental Health Research*, *16*(5), 385–390. https://doi.org/10.1080/09603120600869448
[85]	Baker, S., Holt, K. E., Clements, A. C. A., Karkey, A., Arjyal, A., Boni, M. F., Dongol, S., Hammond, N., Koirala, S., Duy, P. T., Nga, T. V. T., Campbell, J. I., Dolecek, C., Basnyat, B., Dougan, G., & Farrar, J. J. (2011). Combined high-resolution genotyping and geospatial analysis reveals modes of endemic urban typhoid fever transmission. *Open Biology*, *1*(2), 110008. https://doi.org/10.1098/rsob.110008
[86]	Bänziger, C., Schertenleib, A., Kunwar, B. M., Bhatta, M. R., & Marks, S. J. (2022). Assessing microbial water quality, users' perceptions and system functionality following a combined water safety intervention in rural Nepal. *Frontiers in Water*, *3*. https://doi.org/10.3389/frwa.2021.750802
[87]	Bhandari, P., Banjara, M. R., Singh, A., Kandel, S., Rawal, D. S., & Pant, B. R. (2021). Water quality status of groundwater and municipal water supply (Tap water) from Bagmati river basin in Kathmandu Valley, Nepal. *Journal of Water Sanitation and Hygiene for Development*, *11*(1), 102–111. https://doi.org/10.2166/washdev.2020.190
[88]	Bhatt, M. (2015). Water quality and flux of Bagmati River within Kathmandu Valley, Nepal. *Surface and Sub-Surface Water in Asia: Issues and Perspectives*, 243–263. https://doi.org/10.3233/978-1-61499-540-1-243
[89]	Bhatt, M. P., & Gardner, K. H. (2009). Variation in DOC and trace metal concentration along the heavily urbanized basin in Kathmandu Valley, Nepal. *Environmental Geology (Berlin)*, *58*(4), 867–876. https://doi.org/10.1007/s00254-008-1562-z
[90]	Bhatt, M. P., Masuzawa, T., Yamamoto, M., & Takeuchi, N. (2007). Chemical characteristics of pond waters within the debris area of Lirung Glacier in Nepal Himalaya. *Journal of Limnology*, *66*(2), 71–80. https://doi.org/10.4081/jlimnol.2007.71
[91]	Bhatt, M. P., & McDowell, W. H. (2007). Evolution of chemistry along the Bagmati drainage network in Kathmandu Valley. *Water, Air & Soil Pollution*, *185*(1), 165–176. https://doi.org/10.1007/s11270-007-9439-4

[92]	Bhatt, M. P., McDowell, W. H., Gardner, K. H., & Hartmann, J. (2014). Chemistry of the heavily urbanized Bagmati River system in Kathmandu Valley, Nepal: Export of organic matter, nutrients, major ions, silica, and metals. *Environmental Earth Sciences*, *71*(2), 911–922. https://doi.org/10.1007/s12665-013-2494-9
[93]	Bhatta, D. R., Bangtrakulnonth, A., Tishyadhigama, P., Saroj, S. D., Bandekar, J. R., Hendriksen, R. S., & Kapadnis, B. P. (2007). Serotyping, PCR, phage-typing and antibiotic sensitivity testing of Salmonella serovars isolated from urban drinking water supply systems of Nepal. *Letters in Applied Microbiology*, *44*(6), 588–594. https://doi.org/10.1111/j.1472-765X.2007.02133.x
[94]	Bhetwal, A., Maharjan, A., Shakya, S., Satyal, D., Ghimire, S., Khanal, P. R., & Parajuli, N. P. (2017). Isolation of potential phages against multidrug-resistant bacterial isolates: Promising agents in the rivers of Kathmandu, Nepal. *BioMed Research International*, *2017*, 1–10. https://doi.org/10.1155/2017/3723254
[95]	Bittner, A., Khayyat, A. M. A., Luu, K., Maag, B., Murcott, S. E., Pinto, P. M., Sagara, J., & Wolfe, A. (2002). Drinking water quality & point-of-use treatment studies in Nepal. *Civil Engineering Practice*, *17*(1), 5–24.
[22]	Burlakoti, N., Upadhyaya, J., Ghimire, N., Bajgai, T. R., Chhetri, A. B., Rawal, D. S., Koirala, N., & Pant, B. R. (2020). Physical, chemical and microbiological characterization of processed drinking water in central Nepal: Current state study. *Journal of Water, Sanitation and Hygiene for Development*, *10*(1), 157–165. https://doi.org/10.2166/washdev.2020.111
[96]	Chalise, B., Paudyal, P., Kunwar, B. B., Bishwakarma, K., Thapa, B., Pant, R. R., & Neupane, B. B. (2023). Water quality and hydrochemical assessments of thermal springs, Gandaki Province, Nepal. *Heliyon*, *9*(6). https://doi.org/10.1016/j.heliyon.2023.e17353
[97]	Chapagain, S. K., Pandey, V. P., Shrestha, S., Nakamura, T., & Kazama, F. (2010). Assessment of deep groundwater quality in Kathmandu Valley using multivariate statistical techniques. *Water, Air & Soil Pollution*, *210*(1), 277–288. https://doi.org/10.1007/s11270-009-0249-8
[98]	Dahal, B. M., Fuerhacker, M., Mentler, A., Karki, K. B., Shrestha, R. R., & Blum, W. E. H. (2008). Arsenic contamination of soils and agricultural plants through irrigation water in Nepal. *Environmental Pollution*, *155*(1), 157–163. https://doi.org/10.1016/j.envpol.2007.10.024
[99]	Dahal, B. M., Fuerhacker, M., Mentler, A., Shrestha, R. R., & Blum, W. E. H. (2008). Screening of arsenic in irrigation water used for vegetable production in Nepal. *Archives of Agronomy and Soil Science*, *54*(1), 41–51. https://doi.org/10.1080/03650340701628197
[100]	Dahal, B. M., Sitaula, B. K., Sharma, S., & Bajracharya, R. M. (2007). Effects of agricultural intensification on the quality of rivers in rural watersheds of Nepal. *Journal of Food, Agriculture and Environment*, *5*(1), 341–347.
[23]	Daniel, D., Diener, A., van de Vossenberg, J., Bhatta, M., & Marks, S. J. (2020). Assessing drinking water quality at the point of collection and within household storage containers in the Hilly rural areas of mid and far-western Nepal. *International Journal of Environmental Research and Public Health*, *17*(7), 2172. https://doi.org/10.3390/ijerph17072172
[101]	Dumaru, B., Kayastha, S. P., & Pandey, V. P. (2021). Spring water assessment for quality and suitability for various uses: The case of Thuligaad watershed, western Nepal. *Environmental Earth Sciences*, *80*(17), 586–586. https://doi.org/10.1007/s12665-021-09826-w

[102]	Futawatari, T., Kashiwazaki, H., Hamai, T., & Shrestha, M. P. (2000). Perceived environmental problems by Nepalese of three ecologically different areas. *Journal of Global Environment Engineering*, *6*, 33–49.
[103]	Gautam, B. (2021). Microbiological quality assessment (including antibiogram and threat assessment) of bottled water. *Food Science & Nutrition*, *9*(4), 1980–1988. https://doi.org/10.1002/fsn3.2164
[104]	Gautam, B., Gyanwali, G., & Ussery, D. (2021). Assessment of bacterial load in Polyethylene Terephthalate (PET) bottled water marketed in Kathmandu Valley, Nepal. *International Journal of Polymer Science*, *2021*. https://doi.org/10.1155/2021/6681249
[105]	Ghezzi, L., Iaccarino, S., Carosi, R., Montomoli, C., Simonetti, M., Paudyal, K. R., Cidu, R., & Petrini, R. (2019). Water quality and solute sources in the Marsyangdi River system of Higher Himalayan range (West-Central Nepal). *Science of the Total Environment*, *677*, 580–589. https://doi.org/10.1016/j.scitotenv.2019.04.363
[106]	Ghimire, G. P. S. (1985). Water pollution: A major crisis in Nepal. *Environmentalist*, *5*(3), 193.
[107]	Ghimire, M., Kayastha, S. P., Regmi, T., & Bhuiyan, C. (2023). Hydro-chemical characterisation and quality assessment of shallow groundwater in parts of the Kathmandu Valley, Nepal. *Physics and Chemistry of the Earth*, *129*, 103349. https://doi.org/10.1016/j.pce.2022.103349
[108]	Ghimire, M., & Regmi, T. (2023). Hydrogeochemical characteristics of Kodku River of Lalitpur District, Nepal. *H2Open Journal*, *6*(2), 188–207. https://doi.org/10.2166/h2oj.2023.008
[109]	Ghimire, M., Regmi, T., Kayastha, S. P., & Bhuiyan, C. (2023). Groundwater quality and community health risk in Lalitpur Metropolitan City, Nepal–a geospatial analysis. *Geocarto International*, *38*(1). https://doi.org/10.1080/10106049.2023.2168069
[110]	Guragai, B., Takizawa, S., Hashimoto, T., & Oguma, K. (2017). Effects of inequality of supply hours on consumers' coping strategies and perceptions of intermittent water supply in Kathmandu Valley, Nepal. *Science of the Total Environment*, *599*, 431–441. https://doi.org/10.1016/j.scitotenv.2017.04.182
[111]	Guzzella, L., Poma, G., De Paolis, A., Roscioli, C., & Viviano, G. (2011). Organic persistent toxic substances in soils, waters and sediments along an altitudinal gradient at Mt. Sagarmatha, Himalayas, Nepal. *Environmental Pollution*, *159*(10), 2552–2564. https://doi.org/10.1016/j.envpol.2011.06.015
[112]	Gyawali, T., Pant, S., Nakamura, K., Komai, T., & Paudel, S. R. (2022). Spatial and temporal distribution of arsenic contamination in groundwater of Nawalparasi-West, Nepal: An investigation with suggested countermeasures for South Asian Region. *Environmental Monitoring and Assessment*, *194*(8), 582–582. https://doi.org/10.1007/s10661-022-10276-5
[113]	Hammoud, A., Leung, J., Tripathi, S., Butler, A., Sule, M., & Templeton, M. (2018). The impact of latrine contents and emptying practices on nitrogen contamination of well water in Kathmandu Valley, Nepal. *AIMS Environmental Science*, *5*(3), 143–153. https://doi.org/10.3934/environsci.2018.3.143
[114]	Haramoto, E., & Kitajima, M. (2017). Quantification and genotyping of Aichi virus 1 in water samples in the Kathmandu Valley, Nepal. *Food and Environmental Virology*, *9*(3), 350–353. https://doi.org/10.1007/s12560-017-9283-7

[115]	Haramoto, E., Yamada, K., & Nishida, K. (2011). Prevalence of protozoa, viruses, coliphages and indicator bacteria in groundwater and river water in the Kathmandu Valley, Nepal. *Transactions of the Royal Society of Tropical Medicine and Hygiene*, *105*(12), 711–716. https://doi.org/10.1016/j.trstmh.2011.08.004
[25]	Haramoto, E. (2018). Detection of waterborne protozoa, viruses, and bacteria in groundwater and other water samples in the Kathmandu Valley, Nepal. *IOP Conference Series: Earth and Environmental Science*, *120*(1), 012004. https://doi.org/10.1088/1755-1315/120/1/012004
[26]	Inoue, D., Hinoura, T., Suzuki, N., Pang, J., Malla, R., Shrestha, S., Chapagain, S. K., Matsuzawa, H., Nakamura, T., Tanaka, Y., Ike, M., Nishida, K., & Sei, K. (2015). High-throughput DNA microarray detection of pathogenic bacteria in shallow well groundwater in the Kathmandu Valley, Nepal. *Current Microbiology*, *70*(1), 43–50. https://doi.org/10.1007/s00284-014-0681-x
[116]	Ito, Y., Shrestha Malla, S., Bhattarai, A. P., Haramoto, E., Shindo, J., & Nishida, K. (2020). Waterborne diarrhoeal infection risk from multiple water sources and the impact of an earthquake. *Journal of Water and Health*, *18*(4), 464–476. https://doi.org/10.2166/wh.2020.223
[117]	Joshi, B., & Devkota, A. (2022). Assessment of the water quality of Ghodaghodi Lake using selected physicochemical parameters. *International Journal of Ecology and Environmental Sciences*, *48*(4), 495–504.
[118]	Kafle, B. K., Sharma, C. M., Gurung, S., Raut, N., Kafle, K. R., Bhatta, R., Tripathee, L., Paudyal, R., Guo, J., Kang, S., & Dahal, B. M. (2023). Hydrogeochemistry of two major mid-hill lentic water bodies for irrigation of the Central Himalaya, Nepal. *Environment and Natural Resources Journal*, *21*(2), 171–185. https://doi.org/10.32526/ennrj/21/202200195
[119]	Kanel, S. R., Malla, G. B., & Choi, H. (2013). Modeling and study of the mechanism of mobilization of arsenic contamination in the groundwater of Nepal in South Asia. *Clean Technologies and Environmental Policy*, *15*(6), 1077–1082. https://doi.org/10.1007/s10098-013-0580-z
[120]	Kannel, P. R., Kanel, S. R., Lee, S., & Li, Rushang. (2011). Chemometrics in assessment of seasonal variation of water quality in fresh water systems. *Environmental Monitoring and Assessment*, *174*(1), 529–545. https://doi.org/10.1007/s10661-010-1476-6
[121]	Kannel, P. R., Lee, S., Kanel, S. R., & Khan, S. P. (2007). Chemometric application in classification and assessment of monitoring locations of an urban river system. *Analytica Chimica Acta*, *582*(2), 390–399. https://doi.org/10.1016/j.aca.2006.09.006
[122]	Kannel, P. R., Lee, S., Kanel, S. R., Khan, S. P., & Lee, Y.-S. (2007). Spatial-temporal variation and comparative assessment of water qualities of urban river system: A case study of the river Bagmati (Nepal). *Environmental Monitoring and Assessment*, *129*(1), 433–459. https://doi.org/10.1007/s10661-006-9375-6
[123]	Kannel, P. R., Lee, S., & Lee, Y.-S. (2008). Assessment of spatial–temporal patterns of surface and ground water qualities and factors influencing management strategy of groundwater system in an urban river corridor of Nepal. *Journal of Environmental Management*, *86*(4), 595–604. https://doi.org/10.1016/j.jenvman.2006.12.021
[124]	Karkey, A., Jombart, T., Walker, A. W., Thompson, C. N., Torres, A., Dongol, S., Tran Vu Thieu, N., Pham Thanh, D., Tran Thi Ngoc, D., Voong Vinh, P., Singer, A. C., Parkhill, J., Thwaites, G.,

Basnyat, B., Ferguson, N., & Baker, S. (2016). The ecological dynamics of fecal contamination and Salmonella Typhi and Salmonella Paratyphi A in municipal Kathmandu drinking water. *PLoS Neglected Tropical Diseases*, *10*(1), 1–18. https://doi.org/10.1371/journal.pntd.0004346

[125] Kelly, L. J. (1990). Expedition to Nepal: Research project aims to prevent waterborne disease. *Journal of Environmental Health*, *53*(3), 41–45.

[126] Khadka, M. S. (1993). The groundwater quality situation in alluvial aquifers on the Kathmandu Valley, Nepal. *AGSO Journal of Australian Geology & Geophysics*, *14*(2), 207–211.

[127] Khadka, U., & Ramanathan, A. (2013). Major ion composition and seasonal variation in the Lesser Himalayan Lake: Case of Begnas Lake of the Pokhara Valley, Nepal. *Arabian Journal of Geosciences*, *6*(11), 4191–4206. https://doi.org/10.1007/s12517-012-0677-4

[128] Khadka, Y. J., Iqbal, M. Z., & De Nault, K. J. (2015). Urban pollution of Bagmati River corridor within the densely populated Kathmandu Valley in Nepal. *Asian Journal of Water, Environment and Pollution*, *12*(4), 43–59. https://doi.org/10.3233/AJW-150017

[129] Khatiwada, N. R., Takizawa, S., Tran, T. V. N., & Inoue, M. (2002). Groundwater contamination assessment for sustainable water supply in Kathmandu Valley, Nepal. *Water Science & Technology*, *46*(9), 147. https://doi.org/10.2166/wst.2002.0226

[130] Koju, N. K., Sherpa, C. D., & Koju, N. P. (2022). Assessment of physico-chemical parameters along with the concentration of heavy metals in the effluents released from different industries in Kathmandu Valley. *Water, Air & Soil Pollution*, *233*(5), 176. https://doi.org/10.1007/s11270-022-05645-2

[131] Küpper, T., Apel, C., Bertsch, D., van der Giet, M., van der Giet, S., Graß, M., Cerfontaine, C., Haunolder, M., Hundt, N., Kühn, C., Morrison, A., Museo, S., Timmermann, L., Wernitz, K., & Jäger, J. (2022). Analysis of local drinking water for fecal contamination in Solu-Khumbu / Mt. Everest region, Nepal. *International Journal of Hygiene & Environmental Health*, *246*, 114043. https://doi.org/10.1016/j.ijheh.2022.114043

[132] Liu, Y., Yao, T., Jiao, N., Kang, S., Huang, S., Li, Q., Wang, K., & Liu, X. (2009). Culturable bacteria in glacial meltwater at 6,350 m on the East Rongbuk Glacier, Mount Everest. *Extremophiles: Life Under Extreme Conditions*, *13*(1), 89–99. https://doi.org/10.1007/s00792-008-0200-8

[133] Liu, Y., Yao, T., Jiao, N., Kang, S., Zeng, Y., & Huang, S. (2006). Microbial community structure in moraine lakes and glacial meltwaters, Mount Everest. *FEMS Microbiology Letters*, *265*(1), 98–105. https://doi.org/10.1111/j.1574-6968.2006.00477.x

[134] Maden, N., Singh, A., Smith, L., Maharjan, M., & Shrestha, S. (2011). Factors associated with arsenicosis and arsenic exposure status in Nepal: Implications from community based study. *Journal of Community Health*, *36*(1), 76–82. https://doi.org/10.1007/s10900-010-9282-1

[135] Maharjan, M., Shrestha, R. R., Ahmad, S. A., Watanabe, C., & Ohtsuka, R. (2006). Prevalence of arsenicosis in Terai, Nepal. *Journal of Health, Population, and Nutrition*, *24*(2), 246–252. https://www.jstor.org/stable/23499363

[136] Maharjan, M., Watanabe, C., Ahmad, S. A., Umezaki, M., & Ohtsuka, R. (2007). Mutual interaction between nutritional status and chronic arsenic toxicity due to groundwater contamination in an area of Terai, lowland Nepal. *Journal of Epidemiology & Community Health*, *61*(5), 389–394. https://doi.org/10.1136/jech.2005.045062

[137]	Maharjan, M., Watanabe, C., Ahmad, Sk. A., & Ohtsuka, R. (2005). Short report: Arsenic contamination in drinking water and skin manifestations in lowland Nepal: The first community-based survey. *American Journal of Tropical Medicine and Hygiene*, *73*(2), 477–479. https://doi.org/10.4269/ajtmh.2005.73.477
[138]	Mahato, S., Mahato, A., Karna, P. K., & Balmiki, N. (2018). Investigating aquifer contamination and groundwater quality in eastern Terai region of Nepal. *BMC Research Notes*, *11*(1), 321. https://doi.org/10.1186/s13104-018-3445-z
[139]	Malla, B., Ghaju Shrestha, G. R., Bhandari, D., Tandukar, S., Shrestha, S., Yoshinaga, H., Inoue, D., Sei, K., Nishida, K., Tanaka, Y., Sherchand, J. B., & Haramoto, E. (2015). Detection of Cryptosporidium, Giardia, fecal indicator bacteria, and total bacteria in commercial jar water in the Kathmandu Valley, Nepal. *Journal of Institute of Medicine Nepal*, *37*(2), 10–15. http://hdl.handle.net/2440/123928
[140]	Malla, B., Ghaju Shrestha, R., Tandukar, S., Bhandari, D., Inoue, D., Sei, K., Tanaka, Y., Sherchand, J. B., & Haramoto, E. (2018a). Identification of human and animal fecal contamination in drinking water sources in the Kathmandu Valley, Nepal, using host-associated Bacteroidales quantitative PCR assays. *Water*, *10*(12). https://doi.org/10.3390/w10121796
[141]	Malla, B., Ghaju Shrestha, R., Tandukar, S., Bhandari, D., Inoue, D., Sei, K., Tanaka, Y., Sherchand, J. B., & Haramoto, E. (2018b). Validation of host-specific Bacteroidales quantitative PCR assays and their application to microbial source tracking of drinking water sources in the Kathmandu Valley, Nepal. *Journal of Applied Microbiology*, *125*(2), 609–619. https://doi.org/10.1111/jam.13884
[142]	Malla, B., Ghaju Shrestha, R., Tandukar, S., Bhandari, D., Thakali, O., Sherchand, J. B., & Haramoto, E. (2019). Detection of pathogenic viruses, pathogen indicators, and fecal-source markers within tanker water and their sources in the Kathmandu Valley, Nepal. *Pathogens*, *8*(2). https://doi.org/10.3390/pathogens8020081
[143]	Malla, B., Ghaju Shrestha, R., Tandukar, S., Sherchand, J. B., & Haramoto, E. (2019). Performance evaluation of human-specific viral markers and application of pepper mild mottle virus and crassphage to environmental water samples as fecal pollution markers in the Kathmandu Valley, Nepal. *Food and Environmental Virology*, *11*(3 pp.274–287), 274–287. https://doi.org/10.1007/s12560-019-09389-x
[144]	Malla-Pradhan, R., Pradhan, B. L., Prasai Joshi, T., & Phoungthong, K. (2022). Water quality assessment through numerical indices in Phewa Lake, Nepal. *International Journal of Environmental Analytical Chemistry*, 1–15. https://doi.org/10.1080/03067319.2022.2145473
[145]	Meierhofer, R., Bänziger, C., Deppeler, S., Kunwar, B. M., & Bhatta, M. (2018). From water source to tap of ceramic filters-factors that influence water quality between collection and consumption in rural households in Nepal. *International Journal of Environmental Research and Public Health*, *15*(11). https://doi.org/10.3390/ijerph15112439
[146]	Merz, J., Nakarmi, G., Shrestha, S., Dahal, B. M., Dongol, B. S., Schaffner, M., Shakya, S., Sharma, S., & Weingartner, R. (2004). Public water sources in rural watersheds of Nepal's Middle Mountains: Issues and constraints. *Environmental Management*, *34*(1), 26–37. https://doi.org/10.1007/s00267-004-0118-6

[147]	Moravek, J. A., Shrestha, M. B., & Yonzon, S. (2019). Baseline biodiversity and physiochemical survey in Parvati Kunda and surrounding area in Rasuwa, Nepal. *Journal of Threatened Taxa*, *11*(6), 13734–13747. https://doi.org/10.11609/jott.4481.11.6.13734-13747
[148]	Mueller, B., & Hug, S. J. (2018). Climatic variations and de-coupling between arsenic and iron in arsenic contaminated ground water in the lowlands of Nepal. *Chemosphere*, *210*, 347–358. https://doi.org/10.1016/j.chemosphere.2018.07.024
[149]	Nakano, A., Kurosawa, K., Shamim, U. Md., & Tani, M. (2014). Geochemical assessment of arsenic contamination in well water and sediments from several communities in the Nawalparasi District of Nepal. *Environmental Earth Sciences*, *72*(9), 3269–3280. https://doi.org/10.1007/s12665-014-3231-8
[150]	Nicholson, K. N., Neumann, K., Dowling, C., Gruver, J., Sherman, H., & Sharma, S. (2018). An assessment of drinking water sources in Sagarmatha National Park (Mt Everest Region), Nepal. *Mountain Research and Development*, *38*(4), 353–363. https://doi.org/10.1659/MRD-JOURNAL-D-17-00024.1
[27]	Pant, B. R. (2011). Ground water quality in the Kathmandu valley of Nepal. *Environmental Monitoring and Assessment*, *178*(1), 477–485. https://doi.org/10.1007/s10661-010-1706-y
[151]	Pant, D. N., Poudyal, N., Kumar Bhattacharya, S., Pant, N. D., & Bhattacharya, S. K. (2016). Bacteriological quality of bottled drinking water versus municipal tap water in Dharan municipality, Nepal. *Journal of Health, Population & Nutrition*, *35*, 1–6.
[152]	Pant, R. R., Bishwakarma, K., Basnet, B. B., Pal, K. B., Karki, L., Dhital, Y. P., Bhatta, Y. R., Pant, B. R., & Thapa, L. B. (2021). Distribution and risk appraisal of dissolved trace elements in Begnas Lake and Rupa Lake, Gandaki Province, Nepal. *SN Applied Sciences*, *3*(5). https://doi.org/10.1007/s42452-021-04516-5
[153]	Pant, R. R., Bishwakarma, K., Kandel, K., Poudel, S., Nepal, J., Neupane, B. B., & Singh, V. B. (2023). Seasonal variations and health risk assessment of trace elements in the Badigad River, lesser Himalayas, Nepal. *Acta Geochimica*, *42*(4), 689–703. https://doi.org/10.1007/s11631-023-00611-z
[154]	Pant, R. R., Bishwakarma, K., Nepal, J., Paudel, S., Chand, M. B., Qaisar, F. U. R., Pal, K. B., Thapa, L. B., & Wang, G. (2021). Seasonal variations and health risk assessment of trace elements in Seti River Basin, Gandaki Province, Nepal. *Bulletin of Environmental Contamination and Toxicology*, *107*(3), 441–448. https://doi.org/10.1007/s00128-021-03288-3
[155]	Pant, R. R., Bishwakarma, K., Rehman Qaiser, F. U., Pathak, L., Jayaswal, G., Sapkota, B., Pal, K. B., Thapa, L. B., Koirala, M., Rijal, K., & Maskey, R. (2021). Imprints of COVID-19 lockdown on the surface water quality of Bagmati river basin, Nepal. *Journal of Environmental Management*, *289*, 112522. https://doi.org/10.1016/j.jenvman.2021.112522
[156]	Pant, R. R., Qaiser, F. U. R., Wang, G., Adhikari, S., Bishwakarma, K., Baral, U., Rimal, B., Bhatta, Y. R., & Rijal, K. (2021). Hydrochemical appraisal and solute acquisitions in Seti River Basin, Central Himalaya, Nepal. *Environmental Monitoring and Assessment*, *193*(10), 656–656. https://doi.org/10.1007/s10661-021-09437-9

[157]	Pant, R. R., Zhang, F., Rehman, F. U., Koirala, M., Rijal, K., & Maskey, R. (2020). Spatiotemporal characterization of dissolved trace elements in the Gandaki River, Central Himalaya Nepal. *Journal of Hazardous Materials*, *389*, 121913. https://doi.org/10.1016/j.jhazmat.2019.121913
[158]	Pantha, K., Acharya, K., Mohapatra, S., Khanal, S., Amatya, N., Ospina-Betancourth, C., Butte, G., Shrestha, S. D., Rajbhandari, P., & Werner, D. (2021). Faecal pollution source tracking in the holy Bagmati River by portable 16S rRNA gene sequencing. *NPJ Clean Water*, *4*(1). https://doi.org/10.1038/s41545-021-00099-1
[28]	Pantha, S., Timilsina, S., Pantha, S., Manjan, S. K., & Maharjan, M. (2022). Water quality index of springs in mid-hill of Nepal. *Environmental Challenges*, *9*, 100658. https://doi.org/10.1016/j.envc.2022.100658
[159]	Pathak, D. R., & Hiratsuka, A. (2010). An investigation of nitrate and iron concentrations and their relationship in shallow groundwater systems of Kathmandu. *Desalination and Water Treatment*, *19*(1), 191–197. https://doi.org/10.5004/dwt.2010.1884
[160]	Paudel, K., & Basi-Chipalu, S. (2022). Microbiological assessment for potable water. *Scientific World*, *15*(15), Article 15. https://doi.org/10.3126/sw.v15i15.45647
[161]	Paudyal, R., Kang, S., Sharma, C. M., Tripathee, L., Huang, J., Rupakheti, D., & Sillanpää, M. (2016). Major ions and trace elements of two selected rivers near Everest region, southern Himalayas, Nepal. *Environmental Earth Sciences*, *75*(1), 46–46. https://doi.org/10.1007/s12665-015-4811-y
[162]	Paudyal, R., Kang, S., Sharma, C. M., Tripathee, L., & Sillanpää, M. (2016). Variations of the physicochemical parameters and metal levels and their risk assessment in urbanized Bagmati River, Kathmandu, Nepal. *Journal of Chemistry*, 1–13. https://doi.org/10.1155/2016/6025905
[163]	Pokhrel, B. M., & Thapa, N. (2004). Prevalence of Aeromonas in different clinical and water samples with special reference to gastroenteritis. *Nepal Medical College Journal*, *6*(2), 139–143.
[164]	Poudel, S., Paudyal, A., Sharma, B. P., Sharma, K., Baral, Y., Adhikari, S., & Hada, M. S. S. (2021). Microbial and physico-chemical quality assessment of rivers of Kathmandu Valley. *Nepal Journal of Biotechnology*, *9*(2), 7–13. https://www.doi.org/10.54796/njb.v9i2.41908
[165]	Pradhan, B., Gruendlinger, R., Fuerhapper, I., Pradhan, P., & Pradhanang, S. (2005). Knowledge of water quality and water borne disease in rural Kathmandu Valley, Nepal. *Aquatic Ecosystem Health & Management*, *8*(3), 277–284. https://doi.org/10.1080/14634980500208176
[166]	Pradhan, S. P., Joshi, P., Poudel, P., Ghimire, A., Chhetri, S., Maharjan, J., Khadgi, N., Poudel, M., Luitel, A., Pandey, B. P., Shah, R. D. T., & Sharma, S. (2022). Long-term assessment of water quality of Kathmandu University Drinking Water Supply Centre, Nepal. *Sustainable Water Resources Management*, *8*(2). https://doi.org/10.1007/s40899-022-00636-x
[29]	Rai, S. K., Ono, K., Yanagida, J. I., Ishiyama-Imura, S., Kurokawa, M., & Rai, C. K. (2012). A large-scale study of bacterial contamination of drinking water and its public health impact in Nepal. *Nepal Medical College Journal*, *14*(3), 234–240.
[167]	Rai, S. K., Ono, K., Yanagida, J. I., Kurokawa, M., & Rai, C. K. (2009). Status of drinking water contamination in Mountain Region, Nepal. *Nepal Medical College Journal*, *11*(4), 281–283.
[168]	Regmi, R. K., Mishra, B. K., Masago, Y., Luo, P., Toyozumi-Kojima, A., & Jalilov, S.-M. (2017). Applying a water quality index model to assess the water quality of the major rivers in the

	Kathmandu Valley, Nepal. *Environmental Monitoring and Assessment*, *189*(8), 382–382. https://doi.org/10.1007/s10661-017-6090-4
[169]	Rupakheti, D., Tripathee, L., Kang, S., Sharma, C. M., Paudyal, R., & Sillanpää, M. (2017). Assessment of water quality and health risks for toxic trace elements in urban Phewa and remote Gosainkunda lakes, Nepal. *Human & Ecological Risk Assessment*, *23*(5), 959–973. https://doi.org/10.1080/10807039.2017.1292117
[170]	Sah, J. P., Sah, S. K., Acharya, P., Pant, D., & Lance, V. A. (2000). Assessment of water pollution in the Narayani River, Nepal. *International Journal of Ecology and Environmental Sciences*, *26*(4), 235–252.
[171]	Sapkota, M., Pant, R. R., Pathak, L., Khanal, B., Shrestha, S., Poudel, B., Poudel, S., Thapa, L. B., Pal, K. B., Bishwakarma, K., & Durdiev, K. (2021). Assessment of water quality using multivariate statistical approaches in Jagadishpur Reservoir, Lumbini Province, Nepal. *Sustainable Water Resources Management*, *7*(5). https://doi.org/10.1007/s40899-021-00559-z
[30]	Sarkar, B., Mitchell, E., Frisbie, S., Grigg, L., Adhikari, S., & Maskey Byanju, R. (2022). Drinking water quality and public health in the Kathmandu Valley, Nepal: Coliform bacteria, chemical contaminants, and health status of consumers. *Journal of Environmental and Public Health*, *2022*, e3895859. https://doi.org/10.1155/2022/3895859
[172]	Shakya, B. M., Nakamura, T., Kamei, T., Shrestha, S. D., & Nishida, K. (2019). Seasonal groundwater quality status and nitrogen contamination in the shallow aquifer system of the Kathmandu Valley, Nepal. *Water*, *11*(10). https://doi.org/10.3390/w11102184
[173]	Sharma, C. M., Kang, S., Tripathee, L., Paudyal, R., & Sillanpää, M. (2021). Major ions and irrigation water quality assessment of the Nepalese Himalayan rivers. *Environment, Development and Sustainability*, *23*(2), 2668–2680. https://doi.org/10.1007/s10668-020-00694-1
[174]	Sharma, C., Sharma, S., Bajracharya, R., Gurung, S., Jüttner, I., Kang, S., Zhang, Q., & Li, Q. (2012). First results on bathymetry and limnology of high-altitude lakes in the Gokyo Valley, Sagarmatha (Everest) National Park, Nepal. *Limnology*, *13*(1), 181–192. https://doi.org/10.1007/s10201-011-0366-0
[175]	Sherchand, J. B., Cross, J. H., Jimba, M., Sherchand, S., & Shrestha, M. P. (1999). Study of Cyclospora cayetanensis in health care facilities, sewage water and green leafy vegetables in Nepal. *The Southeast Asian Journal of Tropical Medicine and Public Health*, *30*(1), 58–63.
[176]	Shrestha, A., Six, J., Dahal, D., Marks, S., & Meierhofer, R. (2020). Association of nutrition, water, sanitation and hygiene practices with children's nutritional status, intestinal parasitic infections and diarrhoea in rural Nepal: A cross-sectional study. *BMC Public Health*, *20*, 1241. https://doi.org/10.1186/s12889-020-09302-3
[177]	Shrestha, B. K., & Shakya, J. (2021). Simple method devised for rapid isolation and identification of Vibrio cholerae from water resources of Sunsari District, Nepal. *Nepal Journal of Biotechnology*, *9*(2), 33–38.
[33]	Shrestha, R. G., Tanaka, Y., Malla, B., Bhandari, D., Tandukar, S., Inoue, D., Sei, K., Sherchand, J. B., & Haramoto, E. (2017). Next-generation sequencing identification of pathogenic bacterial genes and their relationship with fecal indicator bacteria in different water sources in the

	Kathmandu Valley, Nepal. *Science of the Total Environment*, *601–602*, 278–284. https://doi.org/10.1016/j.scitotenv.2017.05.105
[178]	Shrestha, R. G., Tanaka, Y., Malla, B., Tandukar, S., Bhandari, D., Inoue, D., Sei, K., Sherchand, J. B., & Haramoto, E. (2018). Development of a quantitative PCR assay for Arcobacter spp. And its application to environmental water samples. *Microbes and Environments*, *33*(3), 309–316. https://doi.org/10.1264/jsme2.ME18052
[179]	Shrestha, R. R., Shrestha, M. P., Upadhyay, N. P., Pradhan, R., Khadka, R., Maskey, A., Maharjan, M., Tuladhar, S., Dahal, B. M., & Shrestha, K. (2003). Groundwater arsenic contamination, its health impact and mitigation program in Nepal. *Journal of Environmental Science & Health, Part A: Toxic/Hazardous Substances & Environmental Engineering*, *38*(1), 185. https://doi.org/10.1081/ESE-120016888
[180]	Shrestha, R. R., Shrestha, M. P., Upadhyay, N. P., Pradhan, R., Khadka, R., Maskey, A., Tuladhar, S., Dahal, B. M., Shrestha, S., & Shrestha, K. B. (2003). Groundwater arsenic contamination in Nepal: A new challenge for water supply sector. *Arsenic Exposure and Health Effects V*, 25–37. https://doi.org/10.1016/B978-044451441-7/50003-8
[34]	Shrestha, S. (2016). Seasonal variation of microbial quality of irrigation water in different sources in the Kathmandu valley, Nepal. *Naresuan University Engineering Journal*, *11*(1), Article 1. https://doi.org/10.14456/nuej.2016.10
[181]	Shrestha, S., Haramoto, E., Malla, R., & Nishida, K. (2015). Risk of diarrhoea from shallow groundwater contaminated with enteropathogens in the Kathmandu Valley, Nepal. *Journal of Water and Health*, *13*(1), 259–260. https://doi.org/10.2166/wh.2014.036
[182]	Shrestha, S., Haramoto, E., Sherchand, J. B., & Junko, S. (2016). Detection of coliform bacteria in irrigation water and on vegetable surfaces in the Kathmandu Valley of Nepal. *Journal of Institute of Medicine Nepal*, *38*(1), 43–47.
[183]	Shrestha, S., Nakamura, T., Magome, J., Aihara, Y., Kondo, N., Haramoto, E., Malla, B., Shindo, J., & Nishida, K. (2018). Groundwater use and diarrhoea in urban Nepal: Novel application of a geostatistical interpolation technique linking environmental and epidemiologic survey data. *International Health*, *10*(5), 324–332. https://doi.org/10.1093/inthealth/ihy037
[184]	Shrestha, S., Nakamura, T., Malla, R., & Nishida, K. (2014). Seasonal variation in the microbial quality of shallow groundwater in the Kathmandu Valley, Nepal. *Water Science & Technology: Water Supply*, *14*(3), 390–397. https://doi.org/10.2166/ws.2013.213
[185]	Shrestha, S., Shrestha, S., Shindo, J., Sherchand, J. B., & Haramoto, E. (2018). Virological quality of irrigation water sources and pepper mild mottle virus and tobacco mosaic virus as index of pathogenic virus contamination level. *Food and Environmental Virology*, *10*(1), 107–120. https://doi.org/10.1007/s12560-017-9324-2
[35]	Silvanus, V., Gupta, R. K., & Shreshta, S. R. (2016). Assessment of water supply and microbial quality of water among schools in the rural Kathmandu Valley, Nepal. *Nepal Medical College Journal*, *18*(1–2), 44–47.
[186]	Spica, N., Green, M., Lown, L., Duwal, R., Fuyal, M., Giri, S., Giri, B., Paetkau, D., & Lamichhane-Khadka, R. (2021). Development of a microbiological paper-based analytical device to detect

	fecal contamination of water in resource-limited settings. *Water, Air & Soil Pollution*, *232*(5), 164–164. https://doi.org/10.1007/s11270-021-05132-0
[187]	Sthapit, N., Malla, B., Ghaju Shrestha, R., Tandukar, S., Sherchand, J. B., Haramoto, E., & Kazama, F. (2020). Investigation of Shiga toxin-producing Escherichia coli in groundwater, river water, and fecal sources in the Kathmandu Valley, Nepal. *Water, Air & Soil Pollution*, *231*(12), 557–557. https://doi.org/10.1007/s11270-020-04920-4
[188]	Subedi, D. P., Tyata, R. B., Khadgi, A., & Wong, C. S. (2012). Physicochemical and microbiological analysis of drinking water treated by using ozone. *Sains Malaysiana*, *41*(6), 739–745.
[189]	Subedi, M., & Aryal, M. (2010). Public perception about drinking jar water and its bacteriological analysis. *Nepal Maedical College Journal, 12*(2), 110–114.
[190]	Subedi, M., Magar, M. G., & Rajbhandari, G. S. (2017). Assessment of quality of underground drinking water: Very near (≤20 meters) and far (>50 meters) from the river. *Nepal Journal of Biotechnology, 5*(1), 21–26.
[191]	Sun, X., Zhang, Q., Li, M., Kandel, K., Rawat, B., Pandey, A., Guo, J., Kang, S., Pant, R. R., Cong, Z., & Zhang, F. (2020). Mercury variation and export in trans-Himalayan rivers: Insights from field observations in the Koshi River. *Science of the Total Environment*, *738*, 139836. https://doi.org/10.1016/j.scitotenv.2020.139836
[192]	Tamrakar, P., Sapkota, K., & Shakya, S. K. (2017). Water quality assessment of city water supply in Kathmandu Valley. *Indian Journal of Environmental Protection*, *37*(4), 290–297.
[193]	Tanaka, Y., Nishida, K., Nakamura, T., Chapagain, S. K., Inoue, D., Sei, K., Mori, K., Sakamoto, Y., & Kazama, F. (2012). Characterization of microbial communities distributed in the groundwater pumped from deep tube wells in the Kathmandu Valley of Nepal. *Journal of Water and Health*, *10*(1), 170–180. https://doi.org/10.2166/wh.2011.086
[194]	Tandukar, S., Sherchand, J. B., Bhandari, D., Sherchan, S. P., Malla, B., Ghaju Shrestha, R., & Haramoto, E. (2018). Presence of human enteric viruses, protozoa, and indicators of pathogens in the Bagmati River, Nepal. *Pathogens*, *7*(2). https://doi.org/10.3390/pathogens7020038
[195]	Tandukar, S., Sherchand, J. B., Karki, S., Malla, B., Ghaju Shrestha, R., Bhandari, D., Thakali, O., & Haramoto, E. (2019). Co-infection by waterborne enteric viruses in children with gastroenteritis in Nepal. *Healthcare (Basel, Switzerland)*, *7*(1). https://doi.org/10.3390/healthcare7010009
[196]	Thakur, J. K., Diwakar, J., & Singh, S. K. (2015). Hydrogeochemical evaluation of groundwater of Bhaktapur Municipality, Nepal. *Environmental Earth Sciences*, *74*(6), 4973–4988. https://doi.org/10.1007/s12665-015-4514-4
[36]	Thakur, J. K., Thakur, R. K., Ramanathan, A., Kumar, M., & Singh, S. K. (2011). Arsenic contamination of groundwater in Nepal: An overview. *Water, 3*(1), 1–20. https://doi.org/10.3390/w3010001
[197]	Thapa, K., Shrestha, S. M., Rawal, D. S., & Pant, B. R. (2019). Quality of drinking water in Kathmandu Valley, Nepal. *Sustainable Water Resources Management*, *5*(4), 1995–2000. https://doi.org/10.1007/s40899-019-00354-x
[198]	Tripathee, L., Kang, S., Sharma, C. M., Rupakheti, D., Paudyal, R., Huang, J., & Sillanpää, M. (2016). Preliminary health risk assessment of potentially toxic metals in surface water of the

	Himalayan rivers, Nepal. *Bulletin of Environmental Contamination and Toxicology*, *97*(6), 855–862. https://doi.org/10.1007/s00128-016-1945-x
[199]	Upadhyaya, N. P., & Roy, N. N. (1982). Studies in river pollution in Kathmandu Valley. *Indian Journal of Environmental Health*, *24*(2), 124–135.
[200]	Uppal, J., Zheng, Q., & Le, X. (2019). Arsenic in drinking water-recent examples and updates from Southeast Asia. *Current Opinion in Environmental Science & Health*, *7*, 126–135. https://doi.org/10.1016/j.coesh.2019.01.004
[201]	Uprety, S., Dangol, B., Nakarmi, P., Dhakal, I., Sherchan, S. P., Shisler, J. L., Jutla, A., Amarasiri, M., Sano, D., & Nguyen, T. H. (2020). Assessment of microbial risks by characterization of Escherichia coli presence to analyze the public health risks from poor water quality in Nepal. *International Journal of Hygiene & Environmental Health*, *226*, 113484. https://doi.org/10.1016/j.ijheh.2020.113484
[202]	van Geen, A., Radloff, K. A., Aziz, Z., Cheng, Z., Huq, M. R., Ahmed, K. M., Weinman, B., Goodbred, S., Jung, H. B., Zheng, Y., Berg, M., Trang, P. T. K., Charlet, L., Metral, J., Tisserand, D., Guillot, S., Chakraborty, S., Gajurel, A. P., & Upreti, B. N. (2008). Comparison of arsenic concentrations in simultaneously-collected ground water and aquifer particles from Bangladesh, India, Vietnam, and Nepal. *Applied Geochemistry*, *23*(11), 3244–3251. https://doi.org/10.1016/j.apgeochem.2008.07.005
[203]	Warner, NathanielR., Levy, J., Harpp, K., & Farruggia, F. (2008). Drinking water quality in Nepal's Kathmandu Valley: A survey and assessment of selected controlling site characteristics. *Hydrogeology Journal*, *16*(2), 321–334. https://doi.org/10.1007/s10040-007-0238-1
[205]	Yadav, I. C., Devi, N. L., Mohan, D., Shihua, Q., & Singh, S. (2014). Assessment of groundwater quality with special reference to arsenic in Nawalparasi district, Nepal using multivariate statistical techniques. *Environmental Earth Sciences*, *72*(1), 259–273. https://doi.org/10.1007/s12665-013-2952-4
[207]	Yadav, I. C., Devi, N. L., & Singh, S. (2015a). Reductive dissolution of iron oxyhydroxides directs ground water arsenic mobilization in the upstream of Ganges River basin, Nepal. *Journal of Geochemical Exploration*, *148*, 150–160. https://doi.org/10.1016/j.gexplo.2014.09.002
[206]	Yadav, I. C., Devi, N. L., & Singh, S. (2015b). Spatial and temporal variation in arsenic in the groundwater of upstream of Ganges River Basin, Nepal. *Environmental Earth Sciences*, *73*(3), 1265–1279. https://doi.org/10.1007/s12665-014-3480-6
[204]	Yadav, I. C., Dhuldhaj, U. P., Mohan, D., & Singh, S. (2011). Current status of groundwater arsenic and its impacts on health and mitigation measures in the Terai basin of Nepal: An overview. *Environmental Reviews*, *19*, 55–67. https://doi.org/10.1139/a11-002
[208]	Yadav, I. C., Singh, S., Linthoingambi Devi, N., Mohan, D., Pahari, M., Singh Tater, P., & Man Shakya, B. (2012). Spatial distribution of arsenic in groundwater of Southern Nepal. *Reviews of Environmental Contamination & Toxicology*, *218*, 125–140. https://doi.org/10.1007/978-1-4614-3137-4_3
